# Analysis of rhizosphere bacterial communities of tobacco resistant and non-resistant to bacterial wilt in different regions

**DOI:** 10.1038/s41598-022-20293-6

**Published:** 2022-10-31

**Authors:** Haoqi Shi, Peiwen Xu, Shengxin Wu, Wen Yu, Yazhi Cheng, Zhihua Chen, Xingyou Yang, Xiangwen Yu, Bingjie Li, Anming Ding, Weifeng Wang, Yuhe Sun

**Affiliations:** 1 Key Laboratory for Tobacco Gene Resources, Tobacco Research Institute of Chinese Academy of Agricultural Sciences, Qingdao, 266101 China; 2 Graduate School of Chinese Academy of Agricultural Sciences, Beijing, 100081 China; 3 Fujian Institute of Tobacco Agricultural Sciences, Fuzhou, 350003 China; 4 Sichuan Tobacco Science Institute, Sichuan Branch of China National Tobacco Corporation, Chengdu, 615000 China

**Keywords:** Microbiology, Sequencing

## Abstract

Tobacco bacterial wilt has seriously affected tobacco production. Ethyl methanesulfonate (EMS) induced tobacco bacterial wilt resistant mutants are important for the control of tobacco bacterial wilt. High-throughput sequencing technology was used to study the rhizosphere bacterial community assemblages of bacterial wilt resistant mutant tobacco rhizosphere soil (namely KS), bacterial wilt susceptible tobacco rhizosphere soil (namely GS) and bulk soil (namely BS) in Xuancheng, Huanxi, Yibin and Luzhou. Alpha analysis showed that the bacterial community diversity and richness of KS and GS in the four regions were not significantly different. However, analysis of intergroup variation in the top 15 bacterial communities in terms of abundance showed that the bacterial communities of KS and GS were significantly different from BS, respectively. In addition, pH, alkali-hydrolysable nitrogen (AN) and soil organic carbon (SOC) were positively correlated with the bacterial community of KS and negatively correlated with GS in the other three regions except Huanxi. Network analysis showed that the three soils in the four regions did not show a consistent pattern of network complexity. PICRUSt functional prediction analysis showed that the COG functions were similar in all samples. All colonies were involved in RNA processing and modification, chromatin structure and dynamics, etc. In conclusion, our experiments showed that rhizosphere bacterial communities of tobacco in different regions have different compositional patterns, which are strongly related to soil factors.

## Introduction

Bacterial wilt is a soil-borne disease caused by *Ralstonia solanacearum*, which is endemic worldwide and threatens many plants of the *Solanaceae*, including tobacco, eggplant and potato et al.^1, 2^. On the other hand, there are numerous microorganisms in the soil, which has a large number of ecological niches for growth and reproduction on different plants^3^. They have formed various complex interactions and symbiotic relationships with plants^4^. Microorganisms are of great help to improve the disease resistance of crops^5, 6^. For example, potato common scab^7^, bacterial wilt^8^ can be suppressed by the pathogens in the soil and thus act as a disease resistant agent. Studies have also shown that higher soil microbial community diversity can also act as a disease suppressor and enable successful colonization by other species^5^, rhizosphere soil of tomatoes resistant to *R. solanacearum* has a higher bacterial community diversity index than that of non-resistant^9^. In theory, the higher the abundance of beneficial microorganisms in soil, the higher the soil quality and the lower disease incidence of plants. In addition to the influence of host factors, soil conditions may also cause differences among plant rhizosphere microorganisms^10, 11^. The pH, effective phosphorus and potassium content of healthy soils were significantly increased compared to *R. solanacearum* infected soils^12^. The study of Zhang et al.^9^ showed that available phosphate (AP) plays a key role in the distribution of bacterial communities and its role was negatively correlated with soil pH, soil organic carbon (SOC), alkali-hydrolysable nitrogen (AN) and available potassium (AK) contents. Wang et al.^12^ found that AN content was positively correlated with the microbial community of bacterial wilt infected soil, suggesting that AN content may be an indicator of soil microbial community abundance. Researchers have increased the abundance and diversity of rhizosphere bacteria and effectively suppressed BW in tomato by applying microbial agents (integrated microbiome agent, IMA)^13^. Therefore, understanding the environment-microbial-host relationship is of positive practical importance for effective disease control.

Our group produced several tobacco mutants resistant to tobacco bacterial wilt through ethylmethane sulfonate (EMS) induction. In this study, we used these disease resistant and susceptible tobaccos to study the bacterial community assemblages in the rhizosphere soil of bacterial wilt resistant tobacco mutant (namely KS), rhizosphere soil of tobacco susceptible to bacterial wilt (namely GS) and bulk soil (namely BS) in four regions of China (Xuancheng, Anhui; Huanxi, Fujian; Luzhou, Sichuan and Yibin, Sichuan). In the study, the bacterial communities of the three types of soils in four regions of China were analyzed using high-throughput sequencing, with the aim of providing additional biological control resources for the control of *R. solanacearum*.

## Materials and methods

### Plant materials

The plant material consisted of Cuibi-1 (CB-1), a tobacco variety susceptible to *R. solanacearum* and KCB-1, a highly *R. solanacearum* resistant tobacco EMS mutant (resistance derived from CB-1). CB-1 is a staple tobacco variety in several regions of China with good leaf quality but susceptible to *R. solanacearum*. In addition, the Yanyan97 (YY97) variety was used as a resistance control. The Institute of Tobacco Research of the Chinese Academy of Agricultural Sciences mutagenized CB-1 with EMS to produce the highly resistant tobacco mutant K486, which was backcrossed with CB-1 for three generations and selfed to produce a tobacco line KCB-1 with similar quality to CB-1. Seeds of CB-1 and KCB-1 were planted in Xuancheng (118° 36′ E, 30° 54′ N), Huanxi (119° 24′ E, 26° 12′ N), Luzhou (105° 24′ E, 28° 54′ N) and Yibin (104° 36′ E, 28° 48′ N), respectively, in March 15, 2021. After 4 months of tobacco growth when it is in flowering stage, CB-1 gradually showed disease symptoms, while KCB-1 had good resistance to bacterial wilt.

### Disease index statistics

Disease index (DI) statistics were based on a scale of 0–9. 0 represented no disease, 1 represents tobacco with pitting damage to the stem, but no visible wilting of the leaves. 3 represents tobacco with damage to the stem of less than 10 cm in length and wilting of the leaves. 5 represents a 10–20 cm long damage to the tobacco stem and a large degree of wilting of the tobacco leaves. 7 represents a 20 cm or more damage to the tobacco stem and almost complete wilting of the leaves. 9 represents complete death of the entire plant. The DI was calculated as DI = [(0 × n1 + 1 × n2 + 3 × n3 + 5 × n4 + 7 × n5 + 9 × n6)/(N × 9)] × 100. n1–n6 represent the number of plants with scores of 0, 1, 3, 5, 7 and 9, respectively, and N represents the total number of plants.

### Determination of soil properties

The soil types of Xuancheng, Luzhou, Huanxi and Yibin are yellow loam, yellow–brown soil, yellow–loam and yellow–brown soil. The pH, AN, AP, AK and SOC were determined for KS, GS and BS in four areas. Soil pH was measured according to the method of Qi et al.^14^. The content of SOC was determined by potassium dichromate titration^15^. AK was determined by sodium bicarbonate method^12^. In addition, the alkaline hydrolysis diffusion method was used to determine the AN content^14^. Determination of AP by sodium bicarbonate leaching and molybdenum lamp anti-colorimetric method^16^.

### Soil sample collection and preparation

**Figure 1:**
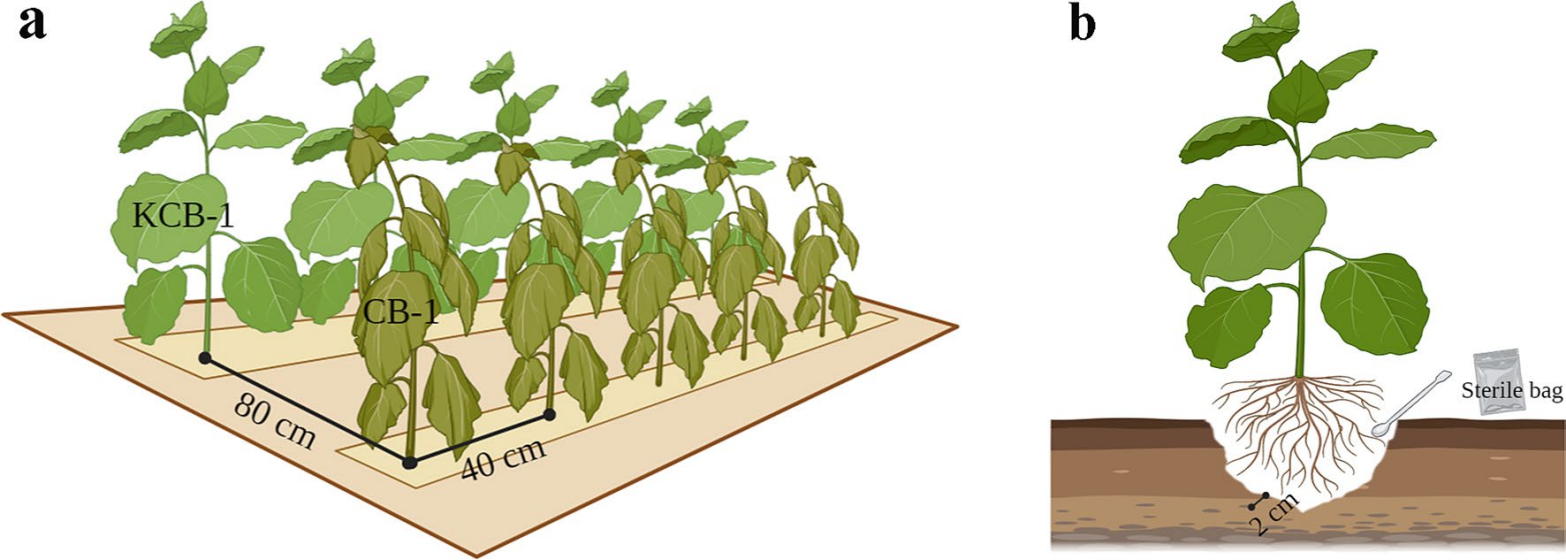
Planting distribution and rhizosphere soil sampling patterns of KCB-1 and CB-1. (**a**) Planting distribution of KCB-1 and CB-1. (**b**) Rhizosphere soil sampling pattern of KCB-1 and CB-1. Images drawing by BioRender (https://biorender.com).

The test plots in these four areas were used exclusively for tobacco cultivation for a long period of time.CB-1 and KCB-1 were grown until July 15, 2021, when significant resistance segregation occurred. At this point, the rhizosphere soil of plants with KCB-1 and CB-1 scores of 0 and 9, respectively, was selected for collection. Five rhizosphere soils of KCB-1 and CB-1 were collected in each of the four areas using the equidistant sampling method. The rhizosphere soil was collected within 2 cm of the root system. The plant spacing was 40 cm for both KCB-1 and CB-1. The row spacing between KCB and CB-1 was 80 cm (Fig. 1a). 100 g of each rhizosphere soil sample was taken using the equidistant sampling method (Fig. 1b). All collected samples were stored in sterile bags and preserved in ice packs and then mailed to Novogene (Tianjin, China) for bacterial 16S sequencing.

### DNA extraction and 16S rRNA gene amplification and sequencing

DNA was extracted from 60 soil samples collected from four areas using the MN NucleoSpin 96 Soi kit. The content and quality of DNA were determined by spectrophotometer (NanoDrop) and agarose gel electrophoresis, respectively. The V3-V4 region of the 16S rRNA gene was amplified using primers 338F and 806R^17^. High-throughput sequencing was performed using the Illumina-MiSeq (Illumina Inc., USA) platform of Shanghai Majorbio Bio-pharm Technology Co., Ltd (Shanghai, China). All sequence data have been deposited in the NCBI Sequence Read Archive database with the registration number PRJNA758569. The software and databases used are included in Table S1.

### Data analysis

Operational taxonomic units (OTU) with 97% similarity were used for sparsity analysis and the diversity index of bacterial communities was calculated. Chao1 and Shannon to assess the abundance and diversity of the rhizosphere bacterial community, respectively. The difference between groups was tested by Student’ *t* test (*p* < 0.05). Principal component analysis (PCA) was performed based on OTU data to calculate the differences of three soil microbial communities in four regions. PERMANOVA was used to analyse the degree of explanation of differences in samples by different groups of factors using Bray–Curtis analysis. The statistical significance of the divisions was also analysed for significance using a permutation test with 999 permutations. The correlations between species were calculated using spearman in the one-way network analysis and screened to leave data with absolute values of correlation coefficients greater than 0.5. Data were plotted using Majorbio Cloud Platform (http://www.majorbio.com) and origin software. Statistical analysis was performed using the SPSSAU website (https://spssau.com/).

### Ethical approval

The authors promise that the experimental research and field studies on plants complies with relevant institutional, national and international guidelines and legislation.

## Results

### High resistance of KCB-1 to bacterial wilt

**Figure 2:**
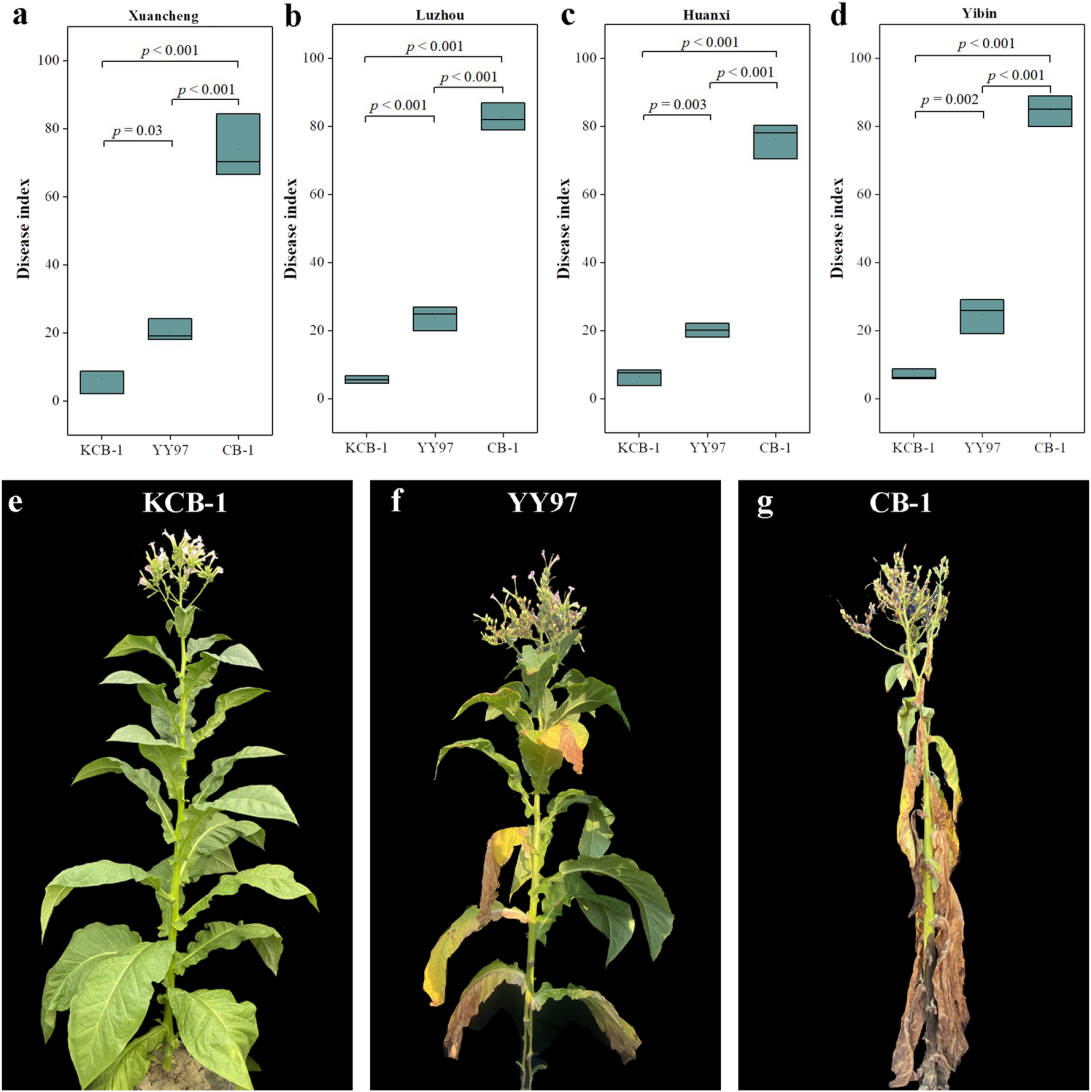
Disease index statistics for KCB-1, CB-1 and YY97. (**a**) Disease index statistics for KCB-1, CB-1 and YY97 in Xuancheng. (**b**) Disease index statistics for KCB-1, CB-1 and YY97 in Luzhou. (**c**) Disease index statistics for KCB-1, CB-1 and YY97 in Huanxi. (**d**) Disease index statistics for KCB-1, CB-1 and YY97 in Yibin. (**e**–**g**) Representative images of the KCB-1, YY97 and CB-1 from the same period. Data were analyzed using one-way ANOVA.

We conducted DI statistical analysis in the field for CB-1, KCB-1 and YY97, which were planted three rows with 20 plants in each row. The results showed that the DI of YY97 was significantly smaller than that of CB-1 (*p* < 0.001) (Fig. 2a–d). the DI of KCB-1 was significantly smaller than that of YY97 and CB-1 (*p* = 0.03, *p* < 0.001) (Fig. 2a–d). Representative images of KCB-1, YY97 and CB-1 from the same period are shown in Fig. 2e–g. The statistical results indicated that KCB-1 is a highly resistant tobacco to bacterial wilt.

### Bacterial diversity assessment of KS, GS and BS in four regions

The low quality reads were filtered to obtain a total of 1,632,420 high quality sequence reads. We refined the dataset by randomly selecting the number of reads in the sample. At 97% similarity, the total number of OTUs for all samples was 12,132. The scalability curves meet the experiment requirements (Fig. S1).

**Figure 3:**
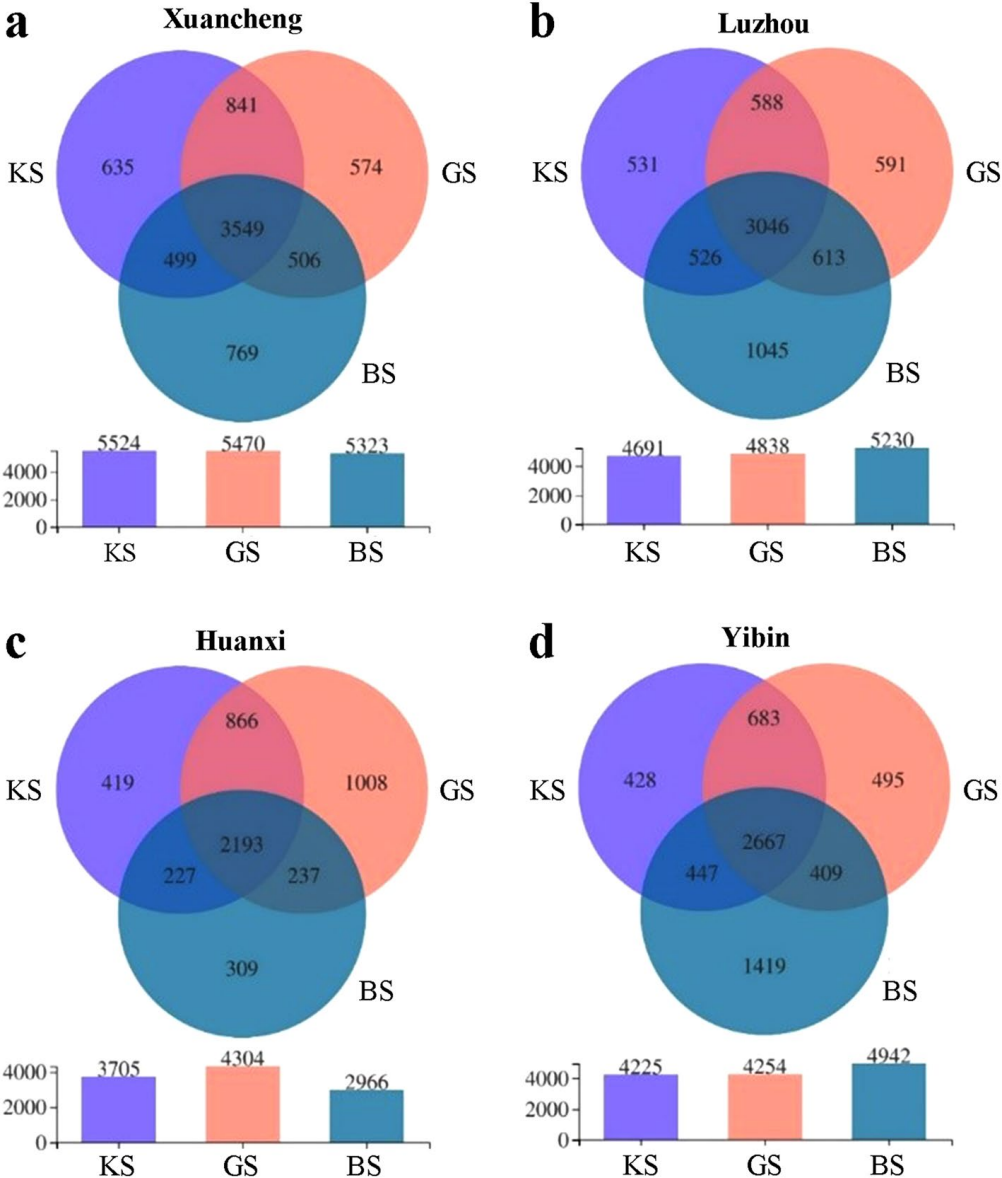
Venn analysis of OTUs among KS, GS and BS in Xuancheng, Luzhou, Huanxi and Yibin. (**a**) Venn analysis of OTUs among KS, GS and BS in Xuancheng. (**b**) Venn analysis of OTUs among KS, GS and BS in Luzhou (**c**) Venn analysis of OTUs among KS, GS and BS in Huanxi. (**d**) Venn analysis of OTUs among KS, GS and BS in Yibin.

Xuancheng had a core OTU count of 3549, with an average of 635, 574 and 769 unique OTUs for KS, GS and BS, respectively (Fig. 3a). Luzhou had a core OTU count of 3046, and KS, GS and BS had an average of 531, 591 and 1045 unique OTUs, respectively (Fig. 3b). Huanxi had a core OTU count of 2193, with KS, GS and BS having an average of 419, 1008 and 309 unique OTUs, respectively (Fig. 3c). Yibin had a core OTU count of 2667, and KS, GS and BS had an average of 428, 495 and 1419 unique OTUs, respectively (Fig. 3d). The total OTUs were divided into 50 phylum and 1309 genera.

### Alpha and PCA analysis of KS, GS and BS in four regions

**Figure 4:**
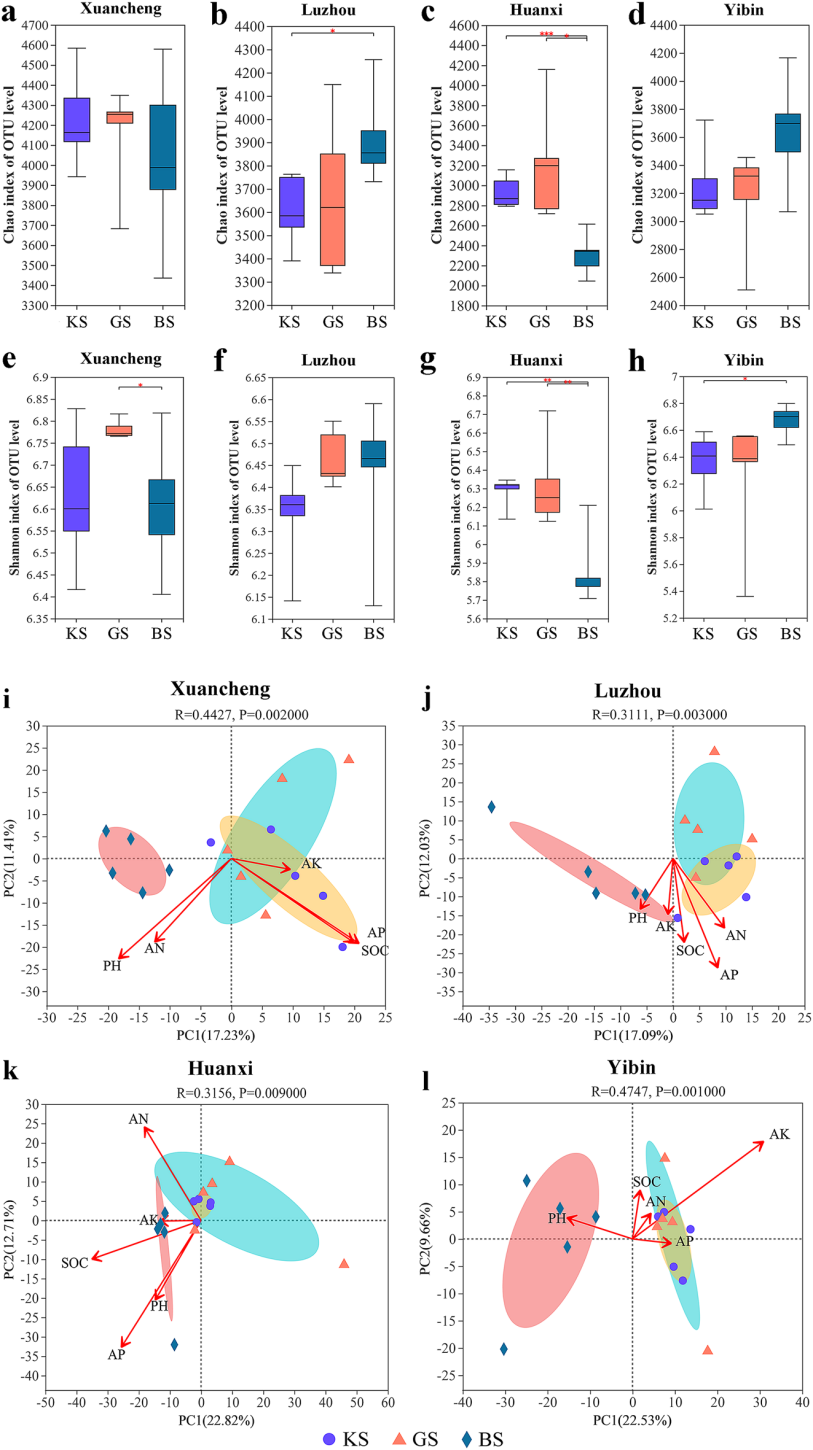
Alpha and PCA analysis of KS, GS and BS in Xuancheng, Luzhou, Huanxi and Yibin. (**a**) Chao1 index of OTU level for KS, GS and BS in Xuancheng. (**b**) Chao1 index of OTU level for KS, GS and BS in Luzhou. (**c**) Chao1 index of OTU level for KS, GS and BS in Huanxi. (**d**) Chao1 index of OTU level for KS, GS and BS in Yibin. (**e**) Shannon index of OTU level for KS, GS and BS in Xuancheng. (**f**) Shannon index of OTU level for KS, GS and BS in Luzhou. (**g**) Shannon index of OTU level for KS, GS and BS in Huanxi. (**h**) Shannon index of OTU level for KS, GS and BS in Yibin. (**i**) PCA of bacterial communities of KS, GS and BS in Xuancheng. (**j**) PCA of bacterial communities of KS, GS and BS in Luzhou. (**k**) PCA of bacterial communities of KS, GS and BS in Huanxi. (**l**) PCA of bacterial communities of KS, GS and BS in Yibin. The data within each group for (**a**–**h**) and (**i**–**l**) were statistically analyzed by Student’ *t *test and PERMANOVA, respectively (*: 0.01 < *p* ≤ 0.05, **: 0.001 < *p* ≤ 0.01, ***: *p* ≤ 0.001).

Alpha diversity refers to the diversity of species in a region. We used Chao1 and Shannon to assess the abundance and diversity of rhizosphere bacterial communities in three soils from four regions, respectively. Chao1 was not significantly different for KS and GS in Xuancheng, Luzhou, Huanxi and Yibin (Student’ *t* test, *p* = 0.65, *p* = 0.72, *p* = 0.32 and *p* = 0.65) (Fig. 4a–d). However, Chao1 of KS and BS were significantly different in Luzhou and Huanxi, respectively (Student’ *t* test, *p* = 0.03, *p* < 0.001) (Fig. 4b,c). In addition, Chao1 was also significantly different between GS and BS in huanxi (*p* = 0.01) (Fig. 4c).

In addition, the shannon of KS and BS, KS and GS of Xuancheng were not significantly different (Student’ *t* test, *p* = 0.86, *p* = 0.07) (Fig. 4e), respectively. However, there was a significant difference in shannon between GS and BS (Student’ *t* test, *p* = 0.04) (Fig. 4e). The shannon of KS and BS, KS and GS, GS and BS in luzhou were not significantly different (Student’ *t* test, *p* = 0.35, *p* = 0.06, *p* = 0.66) (Fig. 4f). The shannon of KS and BS, GS and BS of Huanxi were significantly different (Student’ *t* test, *p* = 0.002, *p* = 0.01) (Fig. 4g), respectively. However, the shannon of GS and KS were not significantly different (Student’ *t* test, *p* = 0.73) (Fig. 4g). The shannon of KS and BS of Yibin were significantly different (Student’ *t* test, *p* = 0.03) (Fig. 4h), respectively. However, the shannon of GS and BS, GS and KS were not significantly different (Student’ *t* test, *p* = 0.10, *p* = 0.66) (Fig. 4h), respectively.

We carried out PCA analysis of the bacterial communities of KS, GS and BS in Xuancheng, Luzhou, Huanxi and Yibin. In addition, we used physiological indicators such as pH, AN, AP, AK and SOC of the three rhizosphere soils in the four regions as covariates for PCA. The physiological indicators of the three rhizosphere soils in the four regions are shown in Fig. S2. The results showed that the bacterial community of BS was significantly different from that of KS and GS in Xuancheng, Luzhou, Huanxi and Yibin, respectively (PERMANOVA, *p* = 0.002, *p* = 0.003, *p* = 0.009 and *p* = 0.001). In contrast, there were no significant differences in bacterial community composition between KS and GS in the three regions, except for Luzhou. (Student’ *t* test, *p* = 0.55, *p* = 0.44 and *p* = 0.13). In addition, to further analyze the relationship between environmental factors, rhizosphere soil samples and rhizosphere bacterial communities in the four regions, we performed an analysis using RDA/CCA. The results showed that pH, AN, AP and SOC had a greater effect on the bacterial community in Xuancheng, while AK had a smaller effect on the bacterial community in Xuancheng (Fig. 4i). In addition, the bacterial community of GS in Xuancheng was negatively correlated with pH, AN and SOC, while the bacterial community of KS was positively correlated with pH, AN, AP and SOC (Fig. 4i). In Luzhou, pH, AN, AP, AK and SOC were all positively correlated with bacterial communities in KS, while they were negatively correlated with GS (Fig. 4j). In Huanxi, pH, AN, AP, AK and SOC had similar effects on KS and GS (Fig. 4k). In Yibin, pH, AN, AK, AP and SOC were positively correlated with the bacterial community of KS, while pH, AN, SOC were negatively correlated with the bacterial community of KS (Fig. 4l). To further investigate the degree of explanation of differences in KS, GS and BS bacterial communities in the four regions by soil physicochemical indicators. We also conducted PERMANOVA analysis for each of the three soils in the four regions. The results showed that AN, AK, SOC, pH and AP explained 0.15 (*p* = 0.002), 0.12 (*p* = 0.002), 0.13 (*p* = 0.002), 0.12 (*p* = 0.002) and 0.09 (*p* = 0.003) of the differences in the rhizosphere bacterial communities of the three soils in the four regions, respectively.

### The major bacterial community composition of KS, GS and BS in the four regions

**Figure 5:**
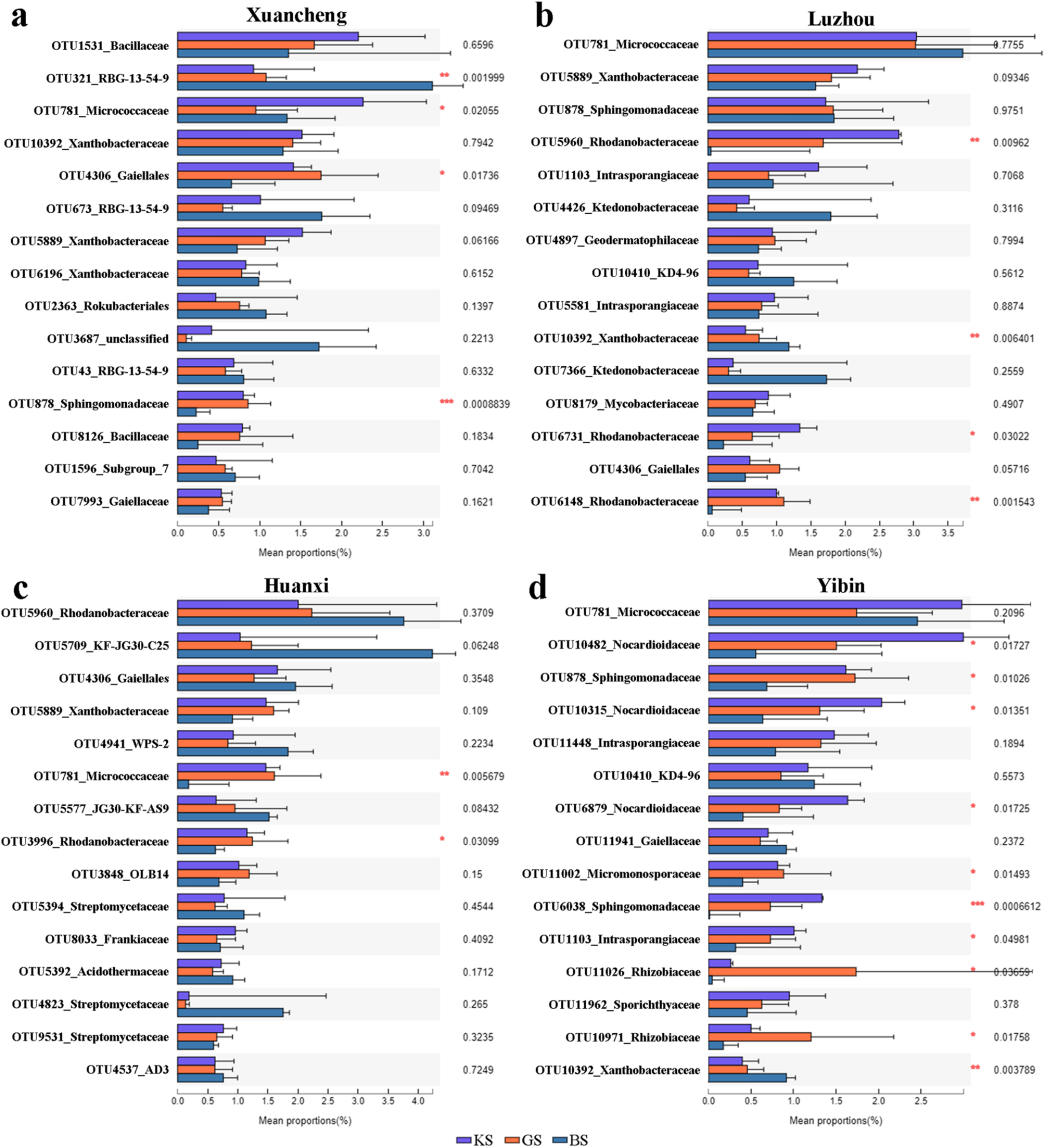
One-way ANOVA and FDR comparison analysis of microbial communities at the OTU level. (**a**) One-way ANOVA and FDR comparison analysis of microbial communities in Xuancheng. (**b**) One-way ANOVA and FDR comparison analysis of microbial communities in Luzhou. (**c**) One-way ANOVA and FDR comparison analysis of microbial communities in Huanxi. (**d)** One-way ANOVA and FDR comparison analysis of microbial communities in Yibin. Data were analyzed by One-way ANOVA (*: 0.01 < *p* ≤ 0.05, **: 0.001 < *p* ≤ 0.01, ***: *p* ≤ 0.001).

We compared the differences between groups for the top 15 most abundant species in each of the four regions. The results showed that among the top 15 species in Xuancheng, OTU321_RBG-13-54-9, OTU781_Micrococcaceae, OTU4306_Gaiellales and OTU878_Sphingomonadaceae had significant differences (One-way ANOVA, *p* = 0.002, *p* = 0.02, *p* = 0.017 and *p* < 0.001), respectively, while the other species did not have significant differences (Fig. 5a). Among the top 15 species in Luzhou, OTU5960_Rhodanobacteraceae, OTU10392_Xanthobacteraceae, OTU6731_Rhodanobacteraceae and OTU6148_Rhodanobacteraceae were significantly different (One-way ANOVA, *p* = 0.01, *p* = 0.006, *p* = 0.03 and *p* = 0.002), respectively, while the other species were not significantly different (Fig. 5b). Among the top 15 species in Huanxi abundance, OTU781_Micrococcaceae and OTU3996_Rhodanobacteraceae were significantly different (One-way ANOVA, *p* = 0.006 and *p* = 0.03), respectively, while the other species were not significantly different (Fig. 5c). Among the top 15 species of Yibin abundance, OTU10482_Nocardioidaceae, OTU878_Sphingomonadaceae, OTU10315_Nocardioidaceae, OTU6879_Nocardioidaceae, OTU11002_Micromonosporaceae, OTU6038_Sphingomonadaceae, OTU1103_Intrasporangiaceae, OTU11026_Rhizobiaceae, OTU10971_Rhizobiaceae, OTU10392_Xanthobacteraceae were significantly different (One-way ANOVA, *p* = 0.017, *p* = 0.01, *p* = 0.014, *p* = 0.017, *p* = 0.015, *p* < 0.001, *p* = 0.049, *p* = 0.037, *p* = 0.018 and *p* = 0.004) and the other species were not significantly different (Fig. 5d).

### Network analysis of KS,GS and BS in four regions

We conducted a one-way correlation network analysis of the top 50 species in terms of total abundance of KS, GS and BS in the four regions. Except for Yibin, the interaction networks of KS in Xuancheng, Luzhou and Huanxi were all larger than that of GS (Fig. S2, Table S2). Overall, the network complexity of KS bacterial communities was higher than that of GS and BS in both Luzhou and Huanxi. In Xuancheng, the network complexity of BS bacterial communities was higher than that of KS and GS. In Yibin, the network complexity of GS bacterial communities was higher than that of KS and BS. In addition to Xuancheng, the beneficial genera present in the KS of Yibin, Luzhou and Huanxi had more interactions. For example, *Micromonospora* and *Sphingomonas* in the KS of Yibin, *Rhodanobacter*, *Sphingomonas* and *Streptomyces* in the KS of Luzhou and *Sphingomonas* in the KS of Huanxi (Table S3).

### PICRUSt functional predictive analysis

PICRUSt function was predicted for 60 samples from four regions. The results showed that the COG functions were relatively similar in all samples. All the colonies were involved in RNA processing and modification, chromatin structure and dynamics, energy production and conversion, cell cycle control, cell division, chromosome partitioning, amino acid transport and metabolism, etc. (Fig. S4).

## Discussion

### Effect of soil physicochemical properties on rhizosphere bacterial communities

The results of the study of bacterial diversity in the four regions using the diversity index Chao1 showed that the bacterial diversity of the three soil types differed between regions. Some areas had the same, while the others showed different diversity of bacterial communities. Therefore, it is a matter of concern whether the regional factor can eliminate the effects associated with differences in plant resistance. In addition, it seems strange that bacterial diversity in GS is instead higher than that in BS in Huanxi. Previous studies have shown that the microbial community diversity in the rhizosphere soil of resistant plants is higher than that of non-resistant plants^8, 9^. This may be due to important differences between BS between regions. Venn analysis of OTUs from three soil types in four regions showed that plant genotype differences leads to colonization and absence of some bacteria, but there is still a portion of bacteria that retains ecological niche homeostasis.

Evidence on whether pH directly or indirectly affects bacterial communities is still unknown^18^. The results of this experiment showed that the pH values of KS and GS were significantly different in all four regions (*p* < 0.05) (Fig. S2a). It is noteworthy that the soil pH of the Huanxi taken was the lowest among the four regions, while the corresponding OTUs of KS, GS and BS in this region were overall at low levels (Fig. 3). Therefore, pH may be one of the factors affecting bacterial diversity. This coincides with previous studies. For example, Rousk et al.^18^ showed that both the relative abundance and diversity of bacteria were positively correlated with pH. In addition, Fierer et al.^19^ showed that the diversity and abundance of soil bacterial communities varied by ecosystem type and that these differences could be explained to a large extent by soil pH. Wang et al.^12^ showed that AN content was positively correlated with the microbial community of bacterial wilt infected soil. It was also shown that AP plays a key role in bacterial community distribution and its role was negatively correlated with soil pH, SOC, AN and AK contents^9^. In addition, Qi et al.^14^ compared the soil chemical properties and microbial network structure of healthy and bacterial wilt infected soils of tobacco. AN, AK and AP contents and soil pH were significantly lower in bacterial wilt infected soils than in healthy soils. There appeared to be a correlation between the physicochemical properties of resistant and susceptible soils and the rhizosphere bacterial community. However, this correlation was not absolute. For example, in this experiment, although the pH of KS was significantly greater than that of GS in all four areas, this was not the case for AN, AP, AK and SOC (Fig. S2).

### Characterization of bacterial communities of resistant and susceptible plants

Plant rhizosphere microbial community composition can vary depending on genotype. For example, there are significant differences in the rhizosphere bacterial communities of different sugarcane varieties^20^. In addition, changes in the location of plant growth can also have an impact on bacterial communities. For example, the effect of maize on the rhizosphere microbial community varies depending on the location of the plant^21^. In addition, plants such as wheat^22^, tobacco^23^ and tomato^24^ exhibit the same situation. It has also been shown that bacterial wilt resistant and susceptible plants have different rhizosphere bacterial communities. For example, the bacterial and fungal community structure of bacterial wilt resistant mulberry is more stable than that of bacterial wilt susceptible mulberry^25^. The correlation regression analysis showed that mulberry bacterial wilt significantly caused loss of soil nutrients, especially organic matter and nitrogen. In this experiment, the four areas had different patterns of species diversity and richness. We have two speculations about this phenomenon: (1) The original bacterial community composition of BS itself is highly variable, and the plant planting does not lead to convergence of microbial networks. (2) Environmental conditions (e.g. temperature and humidity) and the physicochemical properties of the soil affect the composition of the microbial community. Our data support significant differences in bacterial community diversity and richness in different regions of BS (Fig. S5). In addition, neither CB-1 nor KCB-1 showed significant differences in the diversity and abundance of rhizosphere bacterial communities (Fig. 4). This seems to contradict previous findings that the diversity of inter-rhizosphere bacterial communities of bacterial wilt resistant and susceptible plants differed. However, it is worth mentioning that KCB-1 and CB-1 belong to a near-isogenic lineage and their own genetic distance is small, so it is not surprising that the diversity and abundance of the overall rhizosphere microbial communities of both are similar. By comparing the bacterial community abundance of KS and GS in the top 15 abundances among the four regions, the results showed that the bacterial community abundance was not consistent between KS and GS (Fig. 5). We therefore speculate that despite the small genetic distance between KCB-1 and CB-1, there are differences in the effects on the more abundant rhizosphere microorganisms.

Previous studies have shown that root secretions play a key role in plant selection of the rhizosphere microbiome^4, 26, 27^. Many secondary metabolites, such as flavonoids^28^, phenolic compounds^29, 30^, coumarins^31–33^, among others, have been shown to have the ability to shape the composition of microbial communities. In our tentative published data, KCB-1 and CB-1 exhibit different metabolic reprogramming of root metabolites after inoculation with *R. solanacearum*^34^. We focused on coumarins. The results showed that the coumarin-like species in the differential metabolites of KCB-1 and CB-1 roots were different. And previous studies have shown that coumarin-like substances have an important role in shaping rhizosphere microorganisms. For example, Stringlis et al.^31^ showed that coumarin-deficient Arabidopsis mutants recruited a diverse set of microbial communities to their rhizosphere. Furthermore, a study by Voges et al.^32^ showed that the abundance of *Pseudomonas* strains was significantly higher in coumarin-deficient Arabidopsis mutants compared to wild-type plants. Therefore, we speculate that the significantly higher coumarin-like content in KCB-1 contributes to shaping specific microbial communities. However, the bacterial communities of KS and GS were not significantly different in the other three regions except Luzhou (Fig. 4i–l). Therefore, whether secondary metabolites of KCB-1 and CB-1 can influence the overall bacterial community composition remains to be investigated.

The more complex the network of interactions between microorganisms, the more they can adapt to changes in the environment. In addition, it has been shown that complex interactions between microorganisms contribute to the suppression of pathogens^35^. Qi et al.^14^ showed that the BS network is more complex and stable than the GS network. This is in general agreement with our experimental results. The complexity of KS network was more complex than GS in Xuancheng, Huanxi and Luzhou. However, this was not the case in Yibin, which may be the result of the influence of multiple factors under soil fertility and environmental conditions. However, it is certain that a good microbial network can reduce the degree of *R. solanacearum* to some extent.

### Role of beneficial bacteria in rhizosphere soil

More beneficial bacteria colonizing the plant rhizosphere seems to be more beneficial for plants. Qi et al.^14^ found that some beneficial bacteria such as *Bacillus* and *Conexibacter* had more negative interactions in healthy soil than in bacterial wilt susceptible soil. Our results show that, with the exception of Xuancheng, the beneficial genera in the top 50 in KS abundance have more interactions than GS (Table S3). These genera with more interactions were shown to be useful in inducing resistance or promoting plant growth. For example, *Micromonospora* can induce resistance to grey mould in tomato^36^. *Sphingomonas* can promote inter-root growth in Arabidopsis^37^. *Streptomyces* can promote tomato growth and regulate defence-related metabolism^38^. *Rhodanobacter* can promote plant root growth^39^.

In addition, some beneficial genera showed different abundance in different regions and even absent or present in very low abundance in some regions. It is evident that in different regions, depending on the soil as well as environmental conditions, the microbial assemblage can show the absence or extremely low abundance of some bacterial species, and this difference in the assemblage might not be correlated with plant resistance. However, this does not negate the fact that resistant plants can have an impact on the colonization of some bacterial species. This difference due to soil factors comes to a large extent from the influence of plants on the bacterial community. After all, the land conditions were consistent before the planting of bacterial wilt resistant and bacterial wilt non-resistant tobacco.

Bacteria in the same environment may be competing for the same resources, may be dependent on each other for survival, or may interact through host-bacteria feedback, which may increase or decrease the ecological niche space for other bacterial species in the host roots^40^. Biological control using this antagonism between microorganisms might be a good option. Currently, most biocontrols are made by beneficial bacteria, which are mostly isolated from the soil or plant rhizosphere^41, 42^. Using these specific antagonistic microorganisms, such as *Bacillus spp*. by producing antimicrobial metabolites, competing and inducing systemic host resistance to the pathogens. In the future, the discovery of bacterial communities with inhibitory effects on tobacco bacterial wilt will be of great benefit for biological control and is one of the directions we need to work on.

## Supplementary Information

Supplementary Figure S1.

Supplementary Figure S2.

Supplementary Figure S3.

Supplementary Figure S4.

Supplementary Figure S5.

Supplementary Table S1.

Supplementary Table S2.

Supplementary Table S3.

## References

[1] Prior P (2016). Genomic and proteomic evidence supporting the division of the plant pathogen *Ralstonia solanacearum* into three species. BMC Genom..

[2] Peeters N, Guidot A, Vailleau F, Valls M (2013). *Ralstonia solanacearum*, a widespread bacterial plant pathogen in the post-genomic era. Mol. Plant Pathol..

[3] Trivedi P, Leach J, Tringe S, Sa T, Singh B (2020). Plant–microbiome interactions: From community assembly to plant health. Nat. Rev. Microbiol..

[4] Zhalnina K (2018). Dynamic root exudate chemistry and microbial substrate preferences drive patterns in rhizosphere microbial community assembly. Nat. Microbiol..

[5] Kwak M-J (2018). Rhizosphere microbiome structure alters to enable wilt resistance in tomato. Nat. Biotechnol..

[6] van Elsas JD (2012). Microbial diversity determines the invasion of soil by a bacterial pathogen. Proc. Natl. Acad. Sci..

[7] Rosenzweig N, Tiedje JM, Quensen JF, Meng Q, Hao JJ (2012). Microbial communities associated with potato common scab-suppressive soil determined by pyrosequencing analyses. Plant Dis..

[8] Shiomi Y, Nishiyama M, Onizuka T, Marumoto T (1999). Comparison of bacterial community structures in the rhizoplane of tomato plants grown in soils suppressive and conducive towards bacterial wilt. Appl. Environ. Microbiol..

[9] Zhang Y, Hu A, Zhou J, Zhang W, Li P (2020). Comparison of bacterial communities in soil samples with and without tomato bacterial wilt caused by *Ralstonia solanacearum* species complex. BMC Microbiol..

[10] Shi S (2015). Successional trajectories of rhizosphere bacterial communities over consecutive seasons. MBio.

[11] Chaparro JM, Badri DV, Vivanco JM (2013). Rhizosphere microbiome assemblage is affected by plant development. ISME J..

[12] Wang R (2017). Microbial community composition is related to soil biological and chemical properties and bacterial wilt outbreak. Sci. Rep..

[13] Zheng X (2020). Effects of a novel bio-organic fertilizer on the composition of rhizobacterial communities and bacterial wilt outbreak in a continuously mono-cropped tomato field. Appl. Soil Ecol..

[14] Qi G, Ma G, Chen S, Lin C, Zhao X (2019). Microbial network and soil properties are changed in bacterial wilt-susceptible soil. Appl. Environ. Microbiol..

[15] Zhang H (2017). Microbial taxa and functional genes shift in degraded soil with bacterial wilt. Sci. Rep..

[16] Yan Q (2011). Determination of available phosphorus in soil by sodium bicarbonate extraction mo-sb anti-spectrophotometry method. Environ. Monit. Assess..

[17] Wang Y (2020). Analysis of bacterial and fungal communities in continuous-cropping ramie (*Boehmeria nivea* L. Gaud) fields in different areas in China. Sci. Rep..

[18] Rousk J (2010). Soil bacterial and fungal communities across a pH gradient in an arable soil. ISME J..

[19] Fierer N, Jackson RB (2006). The diversity and biogeography of soil bacterial communities. Proc. Natl. Acad. Sci. USA.

[20] Hamonts K (2018). Field study reveals core plant microbiota and relative importance of their drivers. Environ. Microbiol..

[21] Walters WA (2018). Large-scale replicated field study of maize rhizosphere identifies heritable microbes. Proc. Natl. Acad. Sci. USA.

[22] Zheng Y (2021). Wheat-root associated prokaryotic community: Interplay between plant selection and location. Plant Soil.

[23] Zheng Y (2022). Effect of bacterial wilt on fungal community composition in rhizosphere soil of tobaccos in tropical yunnan. Plant Pathol. J..

[24] Lee CG (2017). Comparison of prokaryotic and eukaryotic communities in soil samples with and without tomato bacterial wilt collected from different fields. Microb. Environ..

[25] Dong Z (2020). The dynamics in rhizosphere microbial communities under bacterial wilt resistance by mulberry genotypes. Arch. Microbiol..

[26] Hu L (2018). Root exudate metabolites drive plant-soil feedbacks on growth and defense by shaping the rhizosphere microbiota. Nat. Commun..

[27] Dennis PG, Miller AJ, Hirsch PR (2010). Are root exudates more important than other sources of rhizodeposits in structuring rhizosphere bacterial communities?. FEMS Microbiol. Ecol..

[28] Kudjordjie EN, Sapkota R, Nicolaisen M (2021). Arabidopsis assemble distinct root-associated microbiomes through the synthesis of an array of defense metabolites. PLoS ONE.

[29] Cotton TEA (2019). Metabolic regulation of the maize rhizobiome by benzoxazinoids. ISME J..

[30] Kudjordjie EN, Sapkota R, Steffensen SK, Fomsgaard IS, Nicolaisen M (2019). Maize synthesized benzoxazinoids affect the host associated microbiome. Microbiome.

[31] Stringlis IA (2018). MYB72-dependent coumarin exudation shapes root microbiome assembly to promote plant health. Proc. Natl. Acad. Sci. USA.

[32] Voges MJEEE, Bai Y, Schulze-Lefert P, Sattely ES (2018). Plant-derived coumarins shape the composition of an Arabidopsis synthetic root microbiome. Proc. Natl. Acad. Sci..

[33] Stassen MJJ, Hsu S-H, Pieterse CMJ, Stringlis IA (2020). Coumarin communication along the microbiome–root–shoot axis. Trends Plant Sci..

[34] Shi H (2022). Metabolomic and transcriptomic analysis of roots of tobacco varieties resistant and susceptible to bacterial wilt. Genomics.

[35] Chaffron S, Rehrauer H, Pernthaler J, von Mering C (2010). A global network of coexisting microbes from environmental and whole-genome sequence data. Genome Res..

[36] Martínez-Hidalgo P, García JM, Pozo MJ (2015). Induced systemic resistance against *Botrytis cinerea* by micromonospora strains isolated from root nodules. Front. Microbiol..

[37] Luo Y (2020). Complete genome sequence of *Sphingomonas* sp. Cra20, a drought resistant and plant growth promoting rhizobacteria. Genomics.

[38] Dias MP (2017). Plant growth and resistance promoted by *Streptomyces* spp. in tomato. Plant Physiol. Biochem..

[39] Zhang Y (2020). Soil inoculation of *Trichoderma asperellum* M45a regulates rhizosphere microbes and triggers watermelon resistance to fusarium wilt. AMB Expr..

[40] Wippel K (2021). Host preference and invasiveness of commensals in the lotus and *Arabidopsis* root microbiota. BioRxiv.

[41] Nion YA, Toyota K (2015). Recent trends in control methods for bacterial wilt diseases caused by *Ralstonia solanacearum*. Microb. Environ..

[42] Kurabachew H, Wydra K (2013). Characterization of plant growth promoting rhizobacteria and their potential as bioprotectant against tomato bacterial wilt caused by *Ralstonia solanacearum*. Biol. Control.

