# Supplementary figures and images for: Analysis of rhizosphere bacterial communities of tobacco resistant and non-resistant to bacterial wilt in different regions
Source: Sci Rep. 2022 Oct 31;12:18309. doi: 10.1038/s41598-022-20293-6 (PMC9622857; doi:10.1038/s41598-022-20293-6)

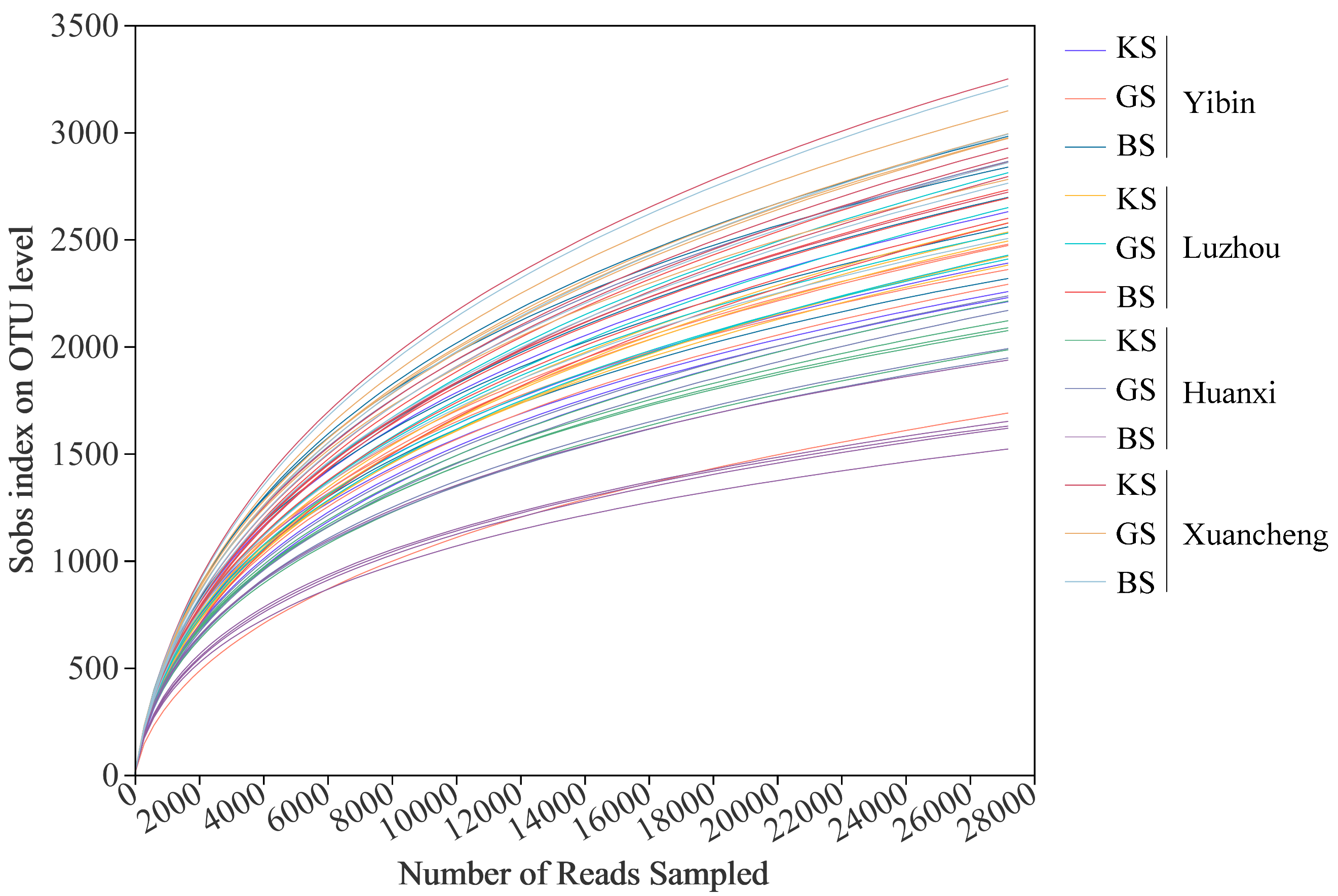


Figure S1. Rarefaction curves of KS, GS and BS of Yibin, Luzhou, Huanxi and Xuancheng.

Supplement: Supplementary file 1 — Supplementary Figure S1. [file 41598_2022_20293_MOESM1_ESM.docx]
