# Supplementary material for: Analysis of rhizosphere bacterial communities of tobacco resistant and non-resistant to bacterial wilt in different regions
Source: Sci Rep. 2022 Oct 31;12:18309. doi: 10.1038/s41598-022-20293-6 (PMC9622857; doi:10.1038/s41598-022-20293-6)
Supplement: Supplementary file 2 — Supplementary Figure S2. [file 41598_2022_20293_MOESM2_ESM.pdf]

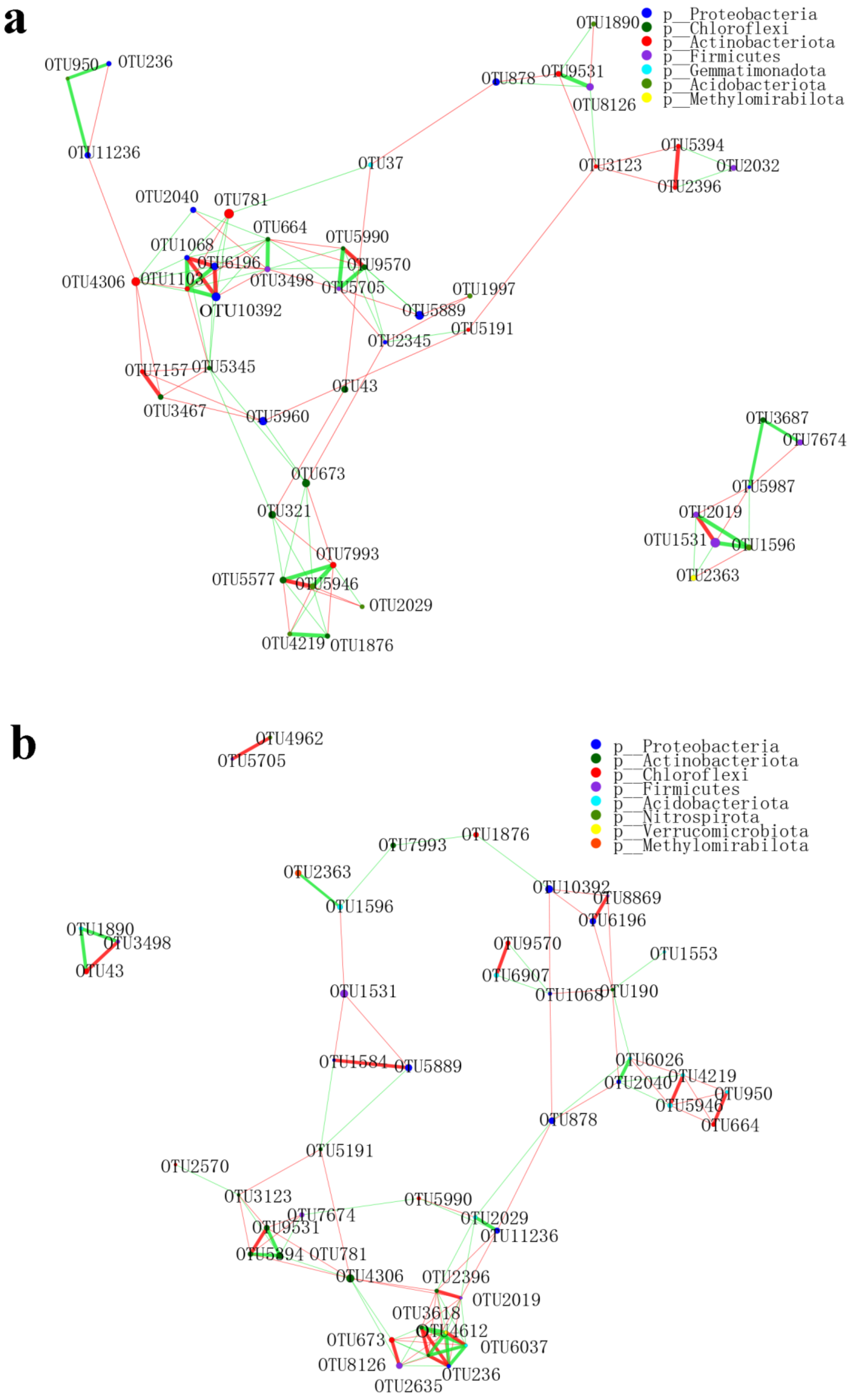
a
p__Proteobacteria
p__Chloroflexi
p__Actinobacteriota
p__Firmicutes
p__Gemmatimonadota
p__Acidobacteriota
p__Methylomirabilota
OTU950
OTU236
OTU11236
OTU1890
OTU878
OTU9531
OTU8126
OTU5394
OTU3123
OTU2032
OTU2396
OTU37
OTU2040
OTU781
OTU664
OTU5990
OTU1068
OTU6196
OTU9570
OTU4306
OTU1103
OTU3498
OTU5705
OTU1997
OTU10392
OTU5889
OTU5191
OTU2345
OTU7157
OTU5345
OTU43
OTU3467
OTU5960
OTU673
OTU321
OTU7993
OTU5577
OTU5946
OTU2029
OTU4219
OTU1876
OTU3687
OTU7674
OTU5987
OTU2019
OTU1531
OTU1596
OTU2363
b
p__Proteobacteria
p__Actinobacteriota
p__Chloroflexi
p__Firmicutes
p__Acidobacteriota
p__Nitrospirota
p__Verrucomicrobiota
p__Methylomirabilota
OTU4962
OTU5705
OTU7993
OTU1876
OTU2363
OTU1596
OTU10392
OTU8869
OTU6196
OTU1890
OTU3498
OTU43
OTU9570
OTU6907
OTU1068
OTU190
OTU1553
OTU1531
OTU1584
OTU5889
OTU6026
OTU2040
OTU4219
OTU950
OTU5946
OTU664
OTU878
OTU5191
OTU2570
OTU3123
OTU7674
OTU9531
OTU5990
OTU2029
OTU11236
OTU5394
OTU781
OTU4306
OTU2396
OTU2019
OTU3618
OTU4612
OTU673
OTU6037
OTU8126
OTU2635
OTU236

c

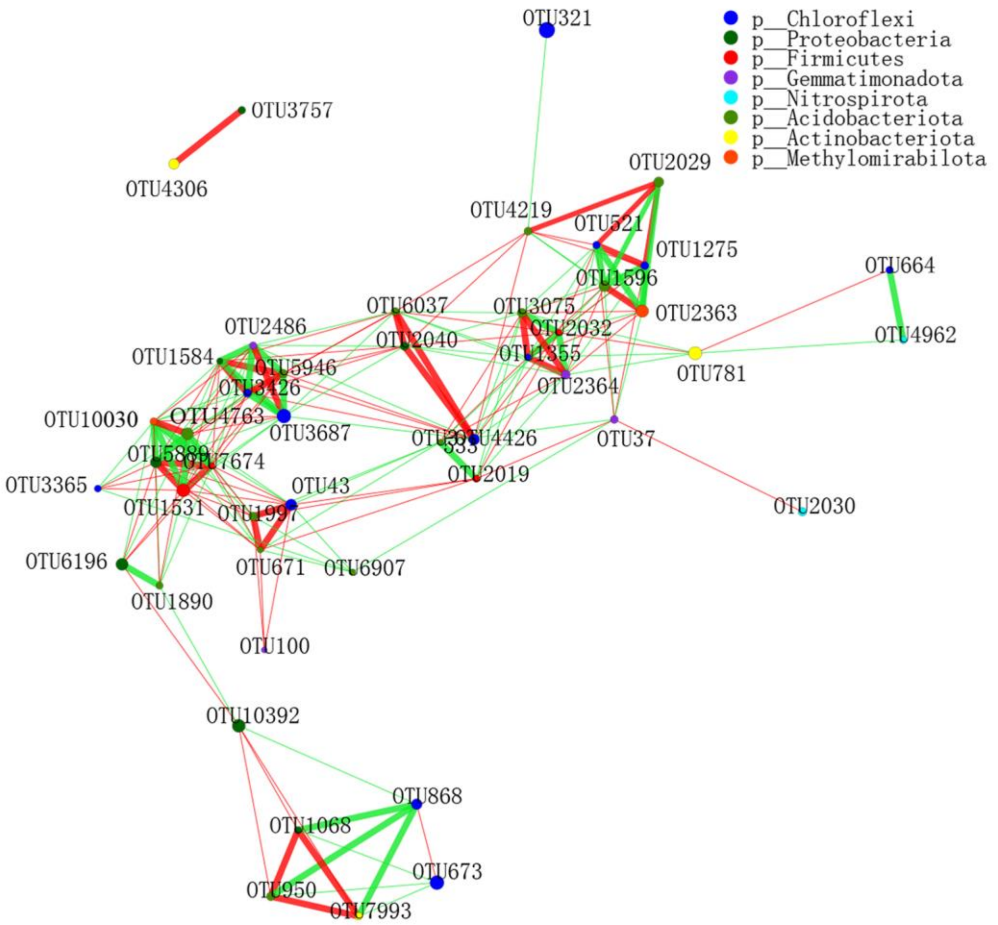
p__Chloroflexi
p__Proteobacteria
p__Firmicutes
p__Gemmatimonadota
p__Nitrospirota
p__Acidobacteriota
p__Actinobacteriota
p__Methylomirabilota
OTU321
OTU3757
OTU4306
OTU2029
OTU4219
OTU521
OTU1275
OTU664
OTU1596
OTU2363
OTU4962
OTU6037
OTU3075
OTU2486
OTU2040
OTU2032
OTU1584
OTU1355
OTU781
OTU5946
OTU2364
OTU3426
OTU10030
OTU4763
OTU3687
OTU4426
OTU37
OTU5889
OTU7674
OTU2019
OTU3365
OTU43
OTU2030
OTU1531
OTU1997
OTU6196
OTU671
OTU6907
OTU1890
OTU100
OTU10392
OTU868
OTU1068
OTU673
OTU950
OTU7993

d

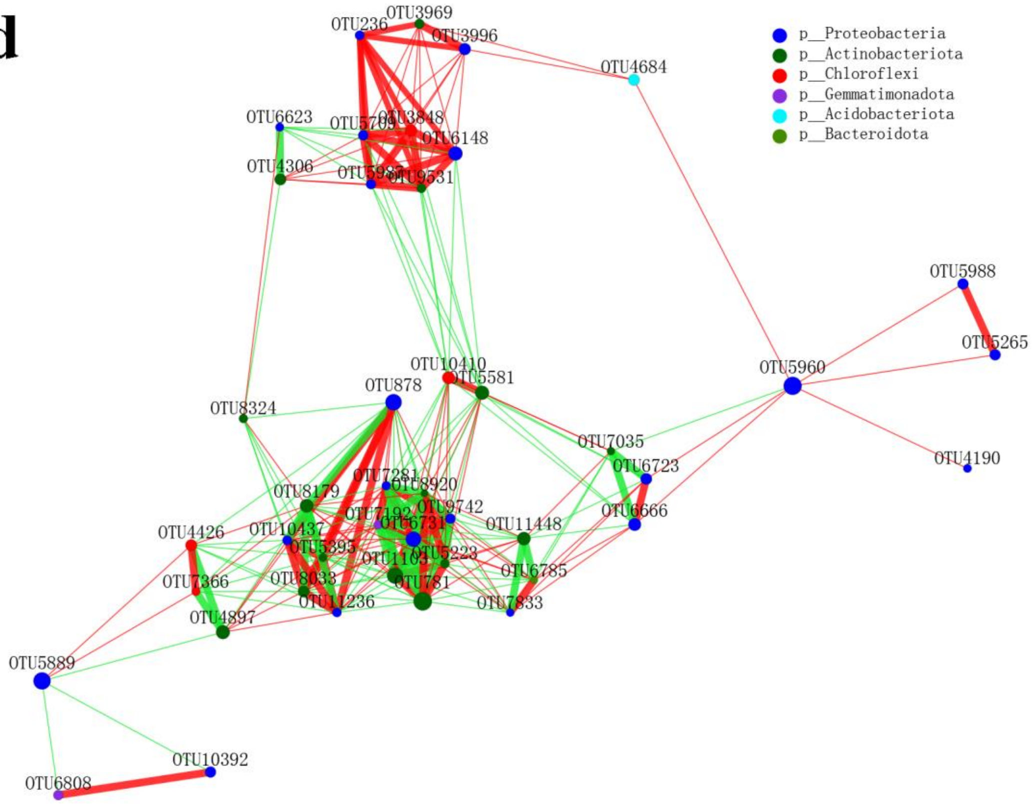
p__Proteobacteria
p__Actinobacteriota
p__Chloroflexi
p__Gemmatimonadota
p__Acidobacteriota
p__Bacteroidota
OTU236
OTU3969
OTU3996
OTU4684
OTU6623
OTU3848
OTU6148
OTU4306
OTU9531
OTU5988
OTU5265
OTU10410
OTU5581
OTU5960
OTU878
OTU8324
OTU7035
OTU6723
OTU4190
OTU7281
OTU8920
OTU8179
OTU9742
OTU6666
OTU11448
OTU4426
OTU10437
OTU5395
OTU5223
OTU6785
OTU7366
OTU8033
OTU781
OTU11236
OTU7833
OTU4897
OTU5889
OTU10392
OTU6808

e

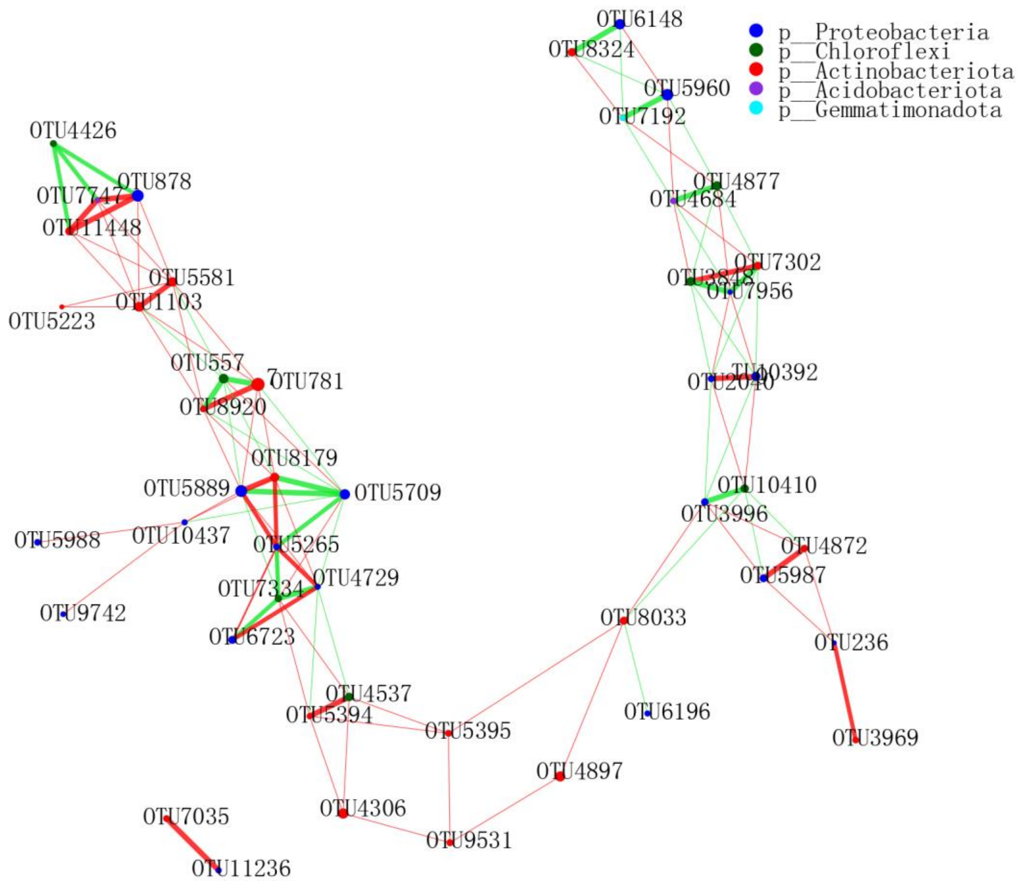
p__Proteobacteria
p__Chloroflexi
p__Actinobacteriota
p__Acidobacteriota
p__Gemmatimonadota
OTU6148
OTU8324
OTU5960
OTU7192
OTU4426
OTU878
OTU11448
OTU4877
OTU4684
OTU5581
OTU1103
OTU5223
OTU7302
OTU7956
OTU781
OTU8920
OTU8179
OTU5889
OTU5709
OTU5988
OTU10437
OTU5265
OTU4729
OTU9742
OTU6723
OTU10410
OTU3996
OTU4872
OTU5987
OTU8033
OTU236
OTU4537
OTU5394
OTU5395
OTU6196
OTU3969
OTU4897
OTU7035
OTU4306
OTU9531
OTU11236

f

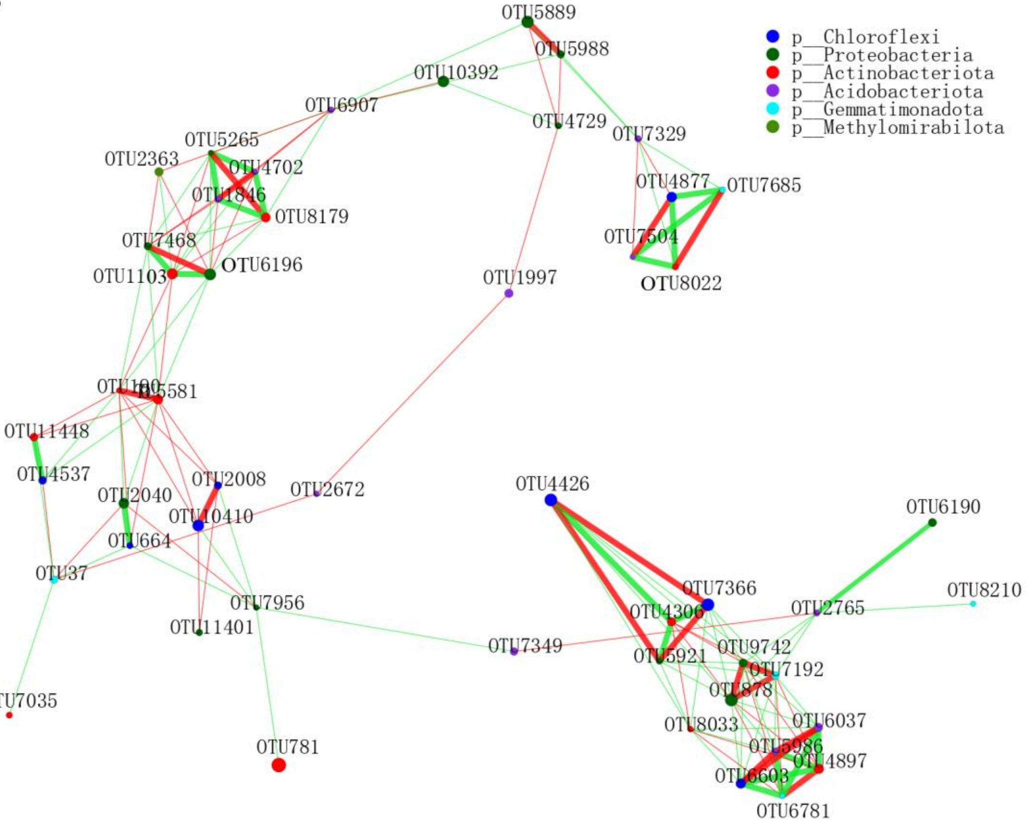
p__Chloroflexi
p__Proteobacteria
p__Actinobacteriota
p__Acidobacteriota
p__Gemmatimonadota
p__Methylomirabilota
OTU5889
OTU5988
OTU10392
OTU6907
OTU4729
OTU7329
OTU5265
OTU2363
OTU4702
OTU1846
OTU8179
OTU4877
OTU7685
OTU7468
OTU7504
OTU1103
OTU6196
OTU8022
OTU1997
OTU5581
OTU11448
OTU4537
OTU2008
OTU2040
OTU2672
OTU10410
OTU4426
OTU6190
OTU664
OTU37
OTU7366
OTU7956
OTU4306
OTU2765
OTU8210
OTU11401
OTU7349
OTU5921
OTU9742
OTU7192
OTU878
OTU7035
OTU8033
OTU6037
OTU5986
OTU4897
OTU781
OTU6603
OTU6781

g

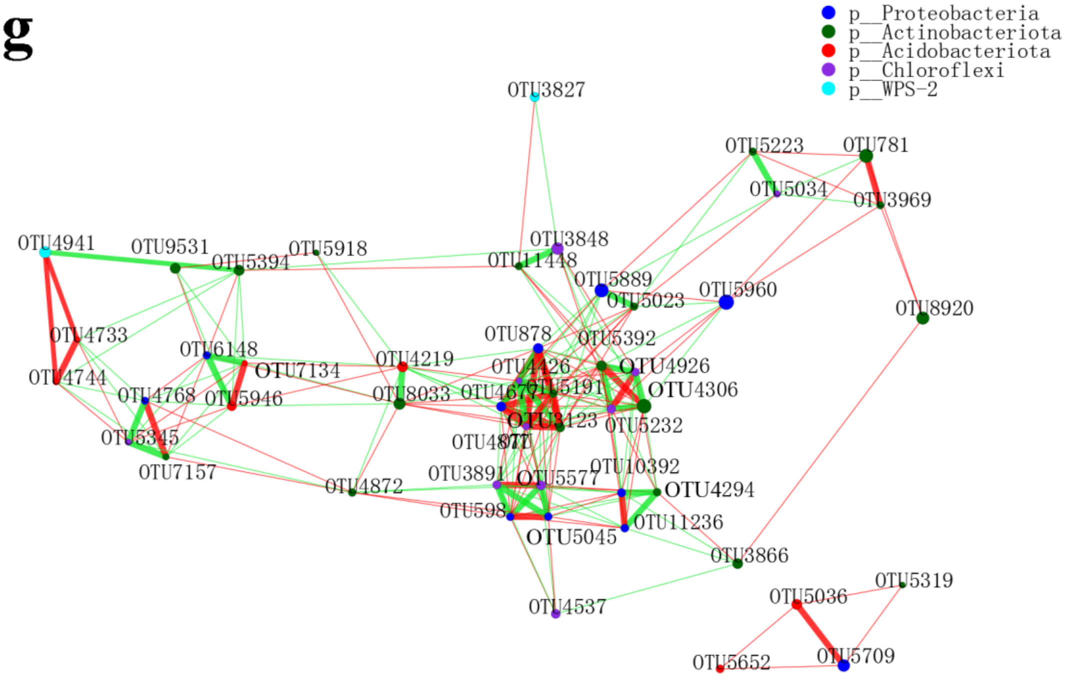
p__Proteobacteria
p__Actinobacteriota
p__Acidobacteriota
p__Chloroflexi
p__WPS-2
OTU3827
OTU5223
OTU781
OTU5034
OTU3969
OTU4941
OTU9531
OTU5394
OTU5918
OTU3848
OTU11448
OTU5889
OTU5023
OTU5960
OTU8920
OTU4733
OTU6148
OTU7134
OTU4219
OTU878
OTU5392
OTU4426
OTU4926
OTU4744
OTU4768
OTU5946
OTU8033
OTU5191
OTU4306
OTU3123
OTU5232
OTU5345
OTU7157
OTU4872
OTU3891
OTU5577
OTU10392
OTU4294
OTU598
OTU11236
OTU5045
OTU3866
OTU4537
OTU5036
OTU5319
OTU5652
OTU5709

h

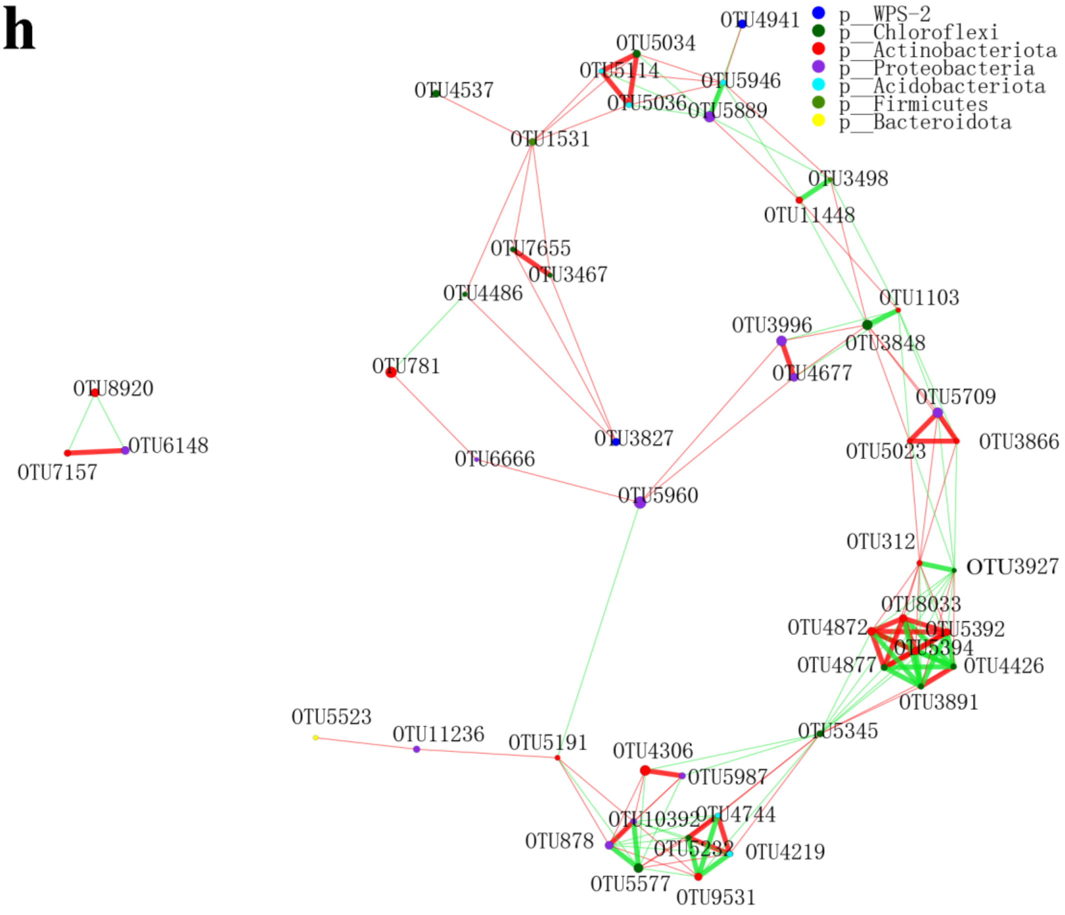
p__WPS-2
p__Chloroflexi
p__Actinobacteriota
p__Proteobacteria
p__Acidobacteriota
p__Firmicutes
p__Bacteroidota
OTU4941
OTU5034
OTU5114
OTU5946
OTU4537
OTU5036
OTU5889
OTU1531
OTU3498
OTU11448
OTU7655
OTU3467
OTU4486
OTU1103
OTU3996
OTU3848
OTU4677
OTU8920
OTU781
OTU5709
OTU6148
OTU3827
OTU5023
OTU3866
OTU7157
OTU6666
OTU5960
OTU312
OTU3927
OTU8033
OTU4872
OTU5392
OTU5394
OTU4877
OTU4426
OTU3891
OTU5523
OTU5345
OTU11236
OTU5191
OTU4306
OTU5987
OTU10392
OTU4744
OTU878
OTU5232
OTU4219
OTU5577
OTU9531

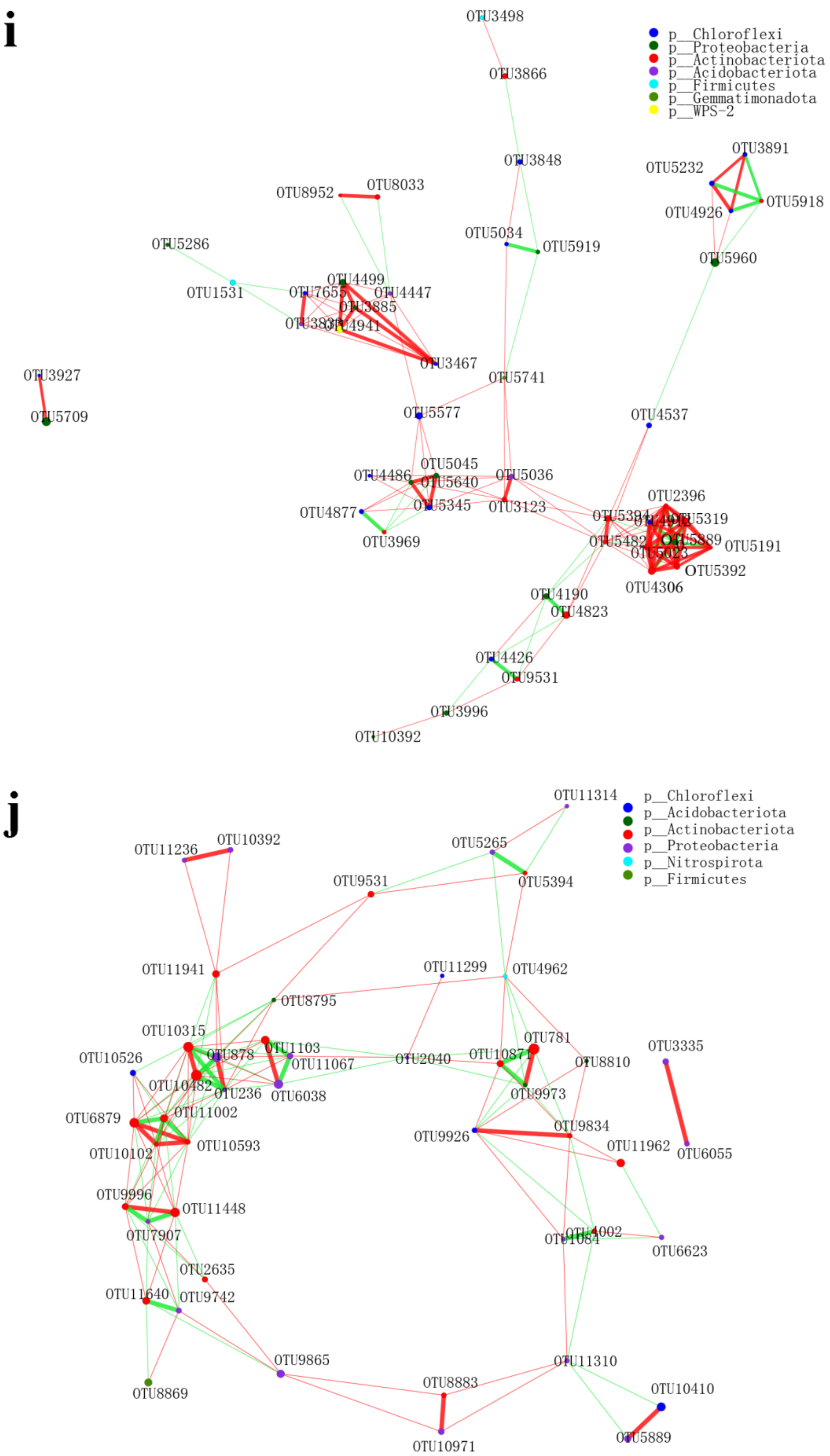

i
p__Chloroflexi
p__Proteobacteria
p__Actinobacteriota
p__Acidobacteriota
p__Firmicutes
p__Gemmatimonadota
p__WPS-2
OTU3498
OTU3866
OTU3848
OTU3891
OTU5232
OTU4926
OTU5918
OTU5960
OTU8952
OTU8033
OTU5034
OTU5919
OTU5286
OTU1531
OTU4499
OTU7655
OTU4447
OTU3885
OTU4941
OTU3467
OTU5741
OTU3927
OTU5709
OTU5577
OTU4537
OTU5045
OTU4486
OTU5640
OTU5036
OTU4877
OTU5345
OTU3123
OTU2396
OTU5319
OTU5191
OTU5482
OTU5889
OTU5392
OTU4306
OTU3969
OTU4190
OTU4823
OTU4426
OTU9531
OTU3996
OTU10392
j
p__Chloroflexi
p__Acidobacteriota
p__Actinobacteriota
p__Proteobacteria
p__Nitrospirota
p__Firmicutes
OTU11314
OTU11236
OTU10392
OTU5265
OTU9531
OTU5394
OTU11941
OTU11299
OTU4962
OTU8795
OTU10315
OTU1103
OTU781
OTU3335
OTU10526
OTU878
OTU11067
OTU2040
OTU10871
OTU8810
OTU10482
OTU236
OTU6038
OTU9973
OTU6879
OTU11002
OTU9834
OTU9926
OTU10593
OTU10102
OTU11962
OTU6055
OTU9996
OTU11448
OTU7907
OTU4002
OTU1084
OTU6623
OTU2635
OTU11640
OTU9742
OTU9865
OTU8883
OTU11310
OTU10410
OTU8869
OTU10971
OTU5889

k

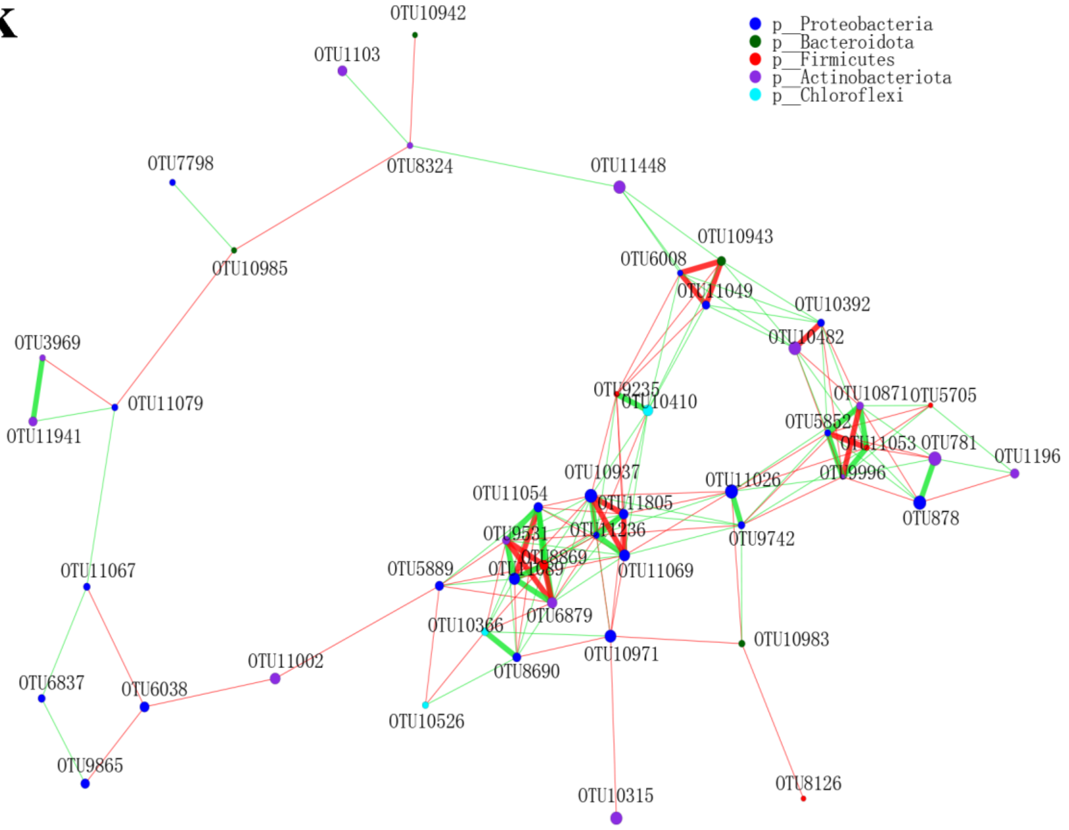
p__Proteobacteria
p__Bacteroidota
p__Firmicutes
p__Actinobacteriota
p__Chloroflexi
OTU10942
OTU1103
OTU8324
OTU7798
OTU11448
OTU10985
OTU10943
OTU6008
OTU11049
OTU10392
OTU3969
OTU10482
OTU11079
OTU11941
OTU9235
OTU10410
OTU10871
OTU5705
OTU5852
OTU11053
OTU781
OTU1196
OTU10937
OTU11026
OTU9996
OTU11054
OTU11805
OTU878
OTU9531
OTU11236
OTU9742
OTU8869
OTU11067
OTU5889
OTU11069
OTU6879
OTU10366
OTU10971
OTU10983
OTU11002
OTU8690
OTU6837
OTU6038
OTU10526
OTU9865
OTU10315
OTU8126

l

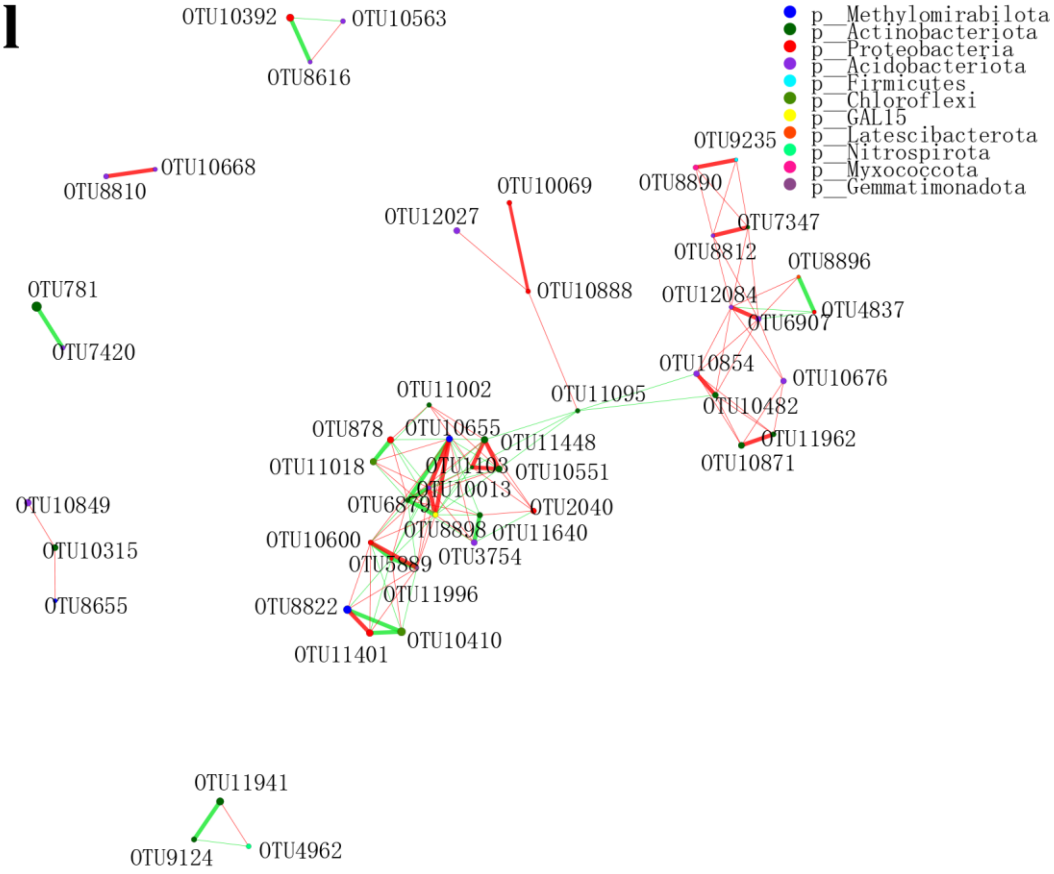
OTU10392
OTU10563
OTU8616
p__Methylomirabilota
p__Actinobacteriota
p__Proteobacteria
p__Acidobacteriota
p__Firmicutes
p__Chloroflexi
p__GAL15
p__Latescibacterota
p__Nitrospirota
p__Myxococcota
p__Gemmatimonadota
OTU9235
OTU10668
OTU8810
OTU8890
OTU10069
OTU12027
OTU7347
OTU8812
OTU8896
OTU781
OTU10888
OTU12084
OTU4837
OTU6907
OTU7420
OTU10854
OTU10676
OTU11002
OTU11095
OTU10482
OTU878
OTU10655
OTU11962
OTU11448
OTU10871
OTU11018
OTU10551
OTU10013
OTU10849
OTU2040
OTU8898
OTU11640
OTU10600
OTU10315
OTU5889
OTU3754
OTU8655
OTU11996
OTU8822
OTU10410
OTU11401
OTU11941
OTU4962
OTU9124

Figure S2. Analysis of OTU level interaction network of KS, GS and HS in Xuancheng, Luzhou, Huanxi and Yibin. a. OTU level interaction network of KS in Xuancheng. b. OTU level interaction network of GS in Xuancheng. c. OTU level interaction network of BS in Xuancheng. d. OTU level interaction network of KS in Luzhou. e. OTU level interaction network of GS in Luzhou. f. OTU level interaction network of BS in Luzhou. g. OTU level interaction network of KS in Huanxi. h. OTU level interaction network of GS in Huanxi. i. OTU level interaction network of BS in Huanxi. j. OTU level interaction network of KS in Yibin. k. OTU level interaction network of GS in Yibin. l. OTU level interaction network of BS in Yibin. Species with correlation coefficient $p \leq 0.05$ are shown in the figure. The size of the nodes in the figure indicates the magnitude of species abundance, and no color is used to indicate the species that are not used. The color of the connecting lines indicates positive and negative correlations, with red indicating positive correlations and green indicating negative correlations. The thickness of the line indicates the magnitude of the correlation coefficient. The thicker the line, the higher the correlation between the species. The more lines, the closer the correlation between the species and other species.
