# Supplementary material for: Analysis of rhizosphere bacterial communities of tobacco resistant and non-resistant to bacterial wilt in different regions
Source: Sci Rep. 2022 Oct 31;12:18309. doi: 10.1038/s41598-022-20293-6 (PMC9622857; doi:10.1038/s41598-022-20293-6)
Supplement: Supplementary file 3 — Supplementary Figure S3. [file 41598_2022_20293_MOESM3_ESM.docx]

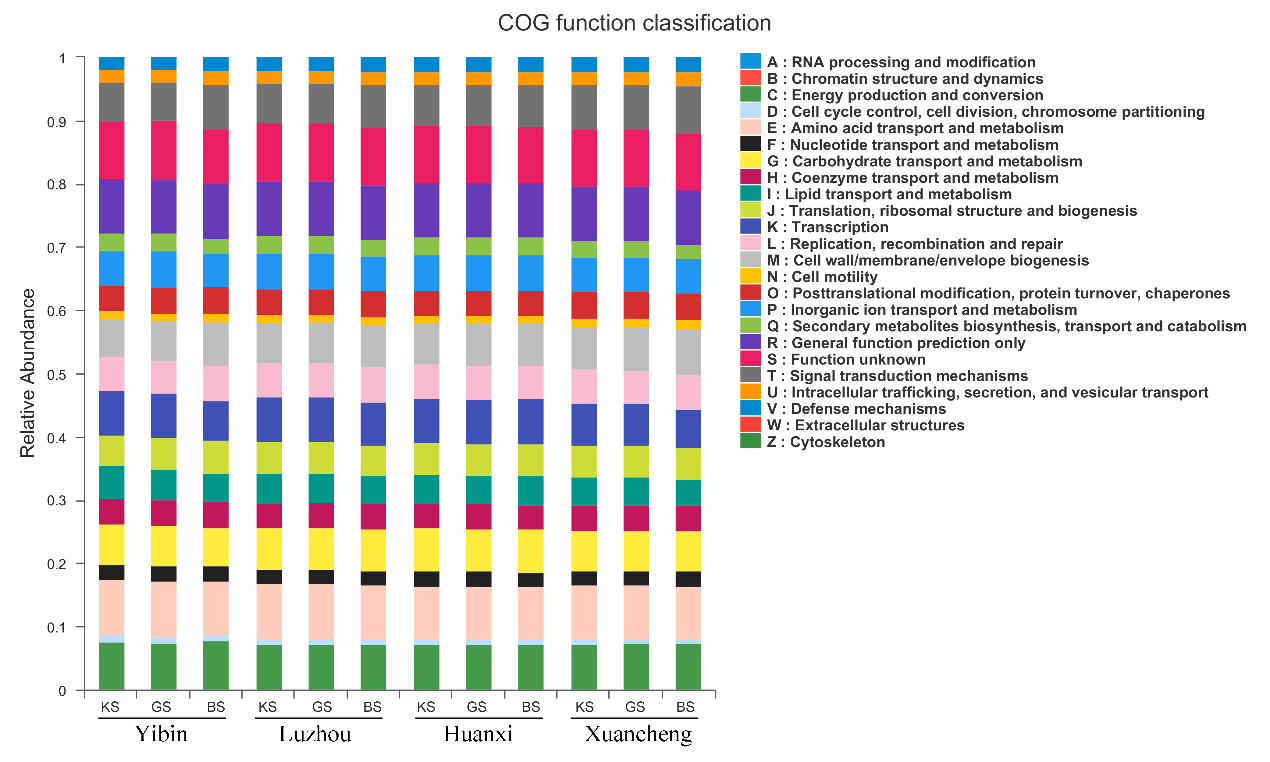


Figure S3. Histogram of COG functional classification statistics of KS, GS and BS in Yibin, Luzhou, Huanxi and Xuancheng.
