# Supplementary material for: Analysis of rhizosphere bacterial communities of tobacco resistant and non-resistant to bacterial wilt in different regions
Source: Sci Rep. 2022 Oct 31;12:18309. doi: 10.1038/s41598-022-20293-6 (PMC9622857; doi:10.1038/s41598-022-20293-6)
Supplement: Supplementary file 4 — Supplementary Figure S4. [file 41598_2022_20293_MOESM4_ESM.docx]

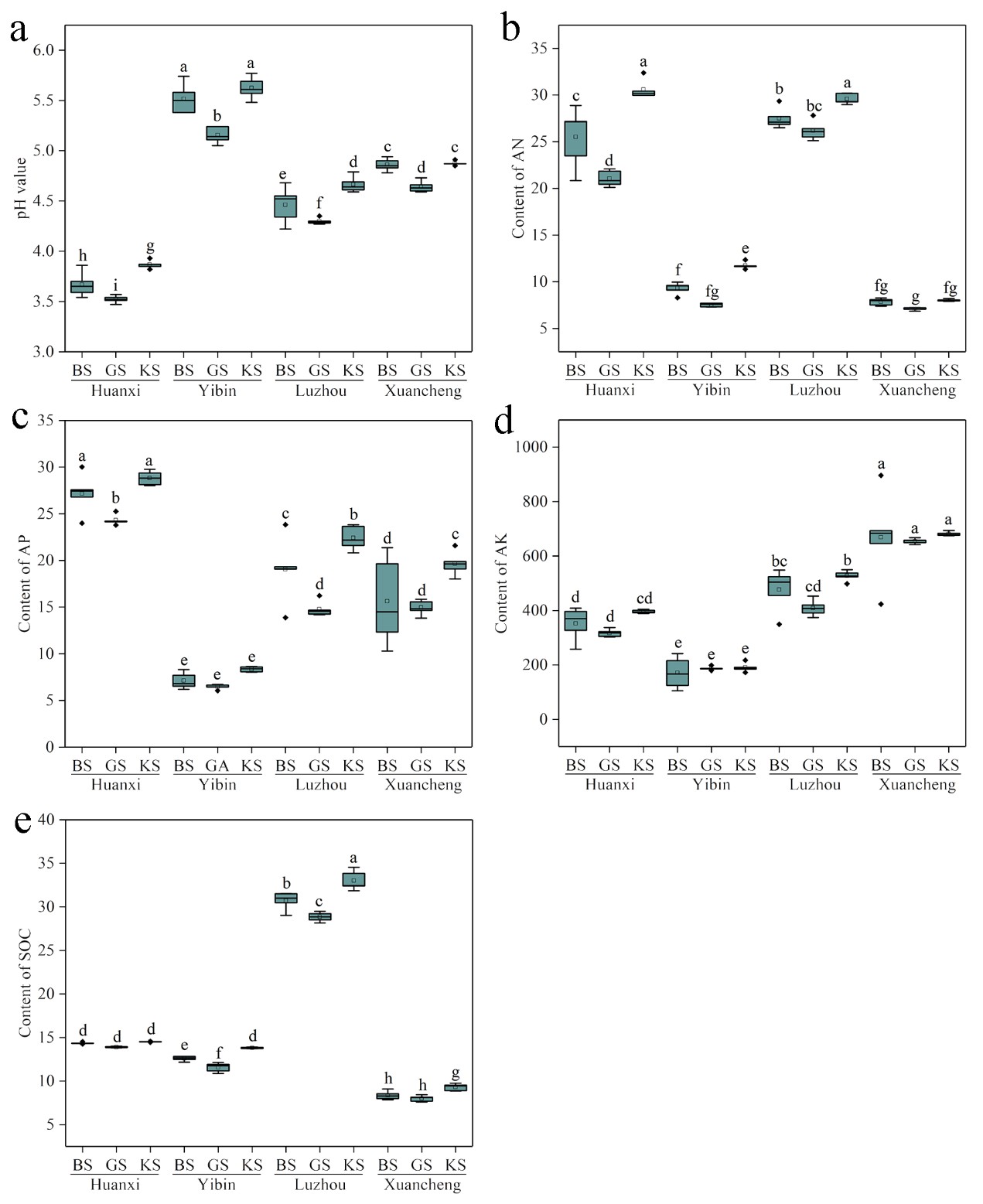


Figure S4. PH, AN, AP, AK and SOC for three soils in four regions. a. PH for three soils in four regions. b. AN for three soils in four regions. c. AP for three soils in four regions. d. AK for three soils in four regions. e. SOC for three soils in four regions. Data were analyzed by one-way ANOVA.
