# Supplementary material for: Analysis of rhizosphere bacterial communities of tobacco resistant and non-resistant to bacterial wilt in different regions
Source: Sci Rep. 2022 Oct 31;12:18309. doi: 10.1038/s41598-022-20293-6 (PMC9622857; doi:10.1038/s41598-022-20293-6)
Supplement: Supplementary file 5 — Supplementary Figure S5. [file 41598_2022_20293_MOESM5_ESM.docx]

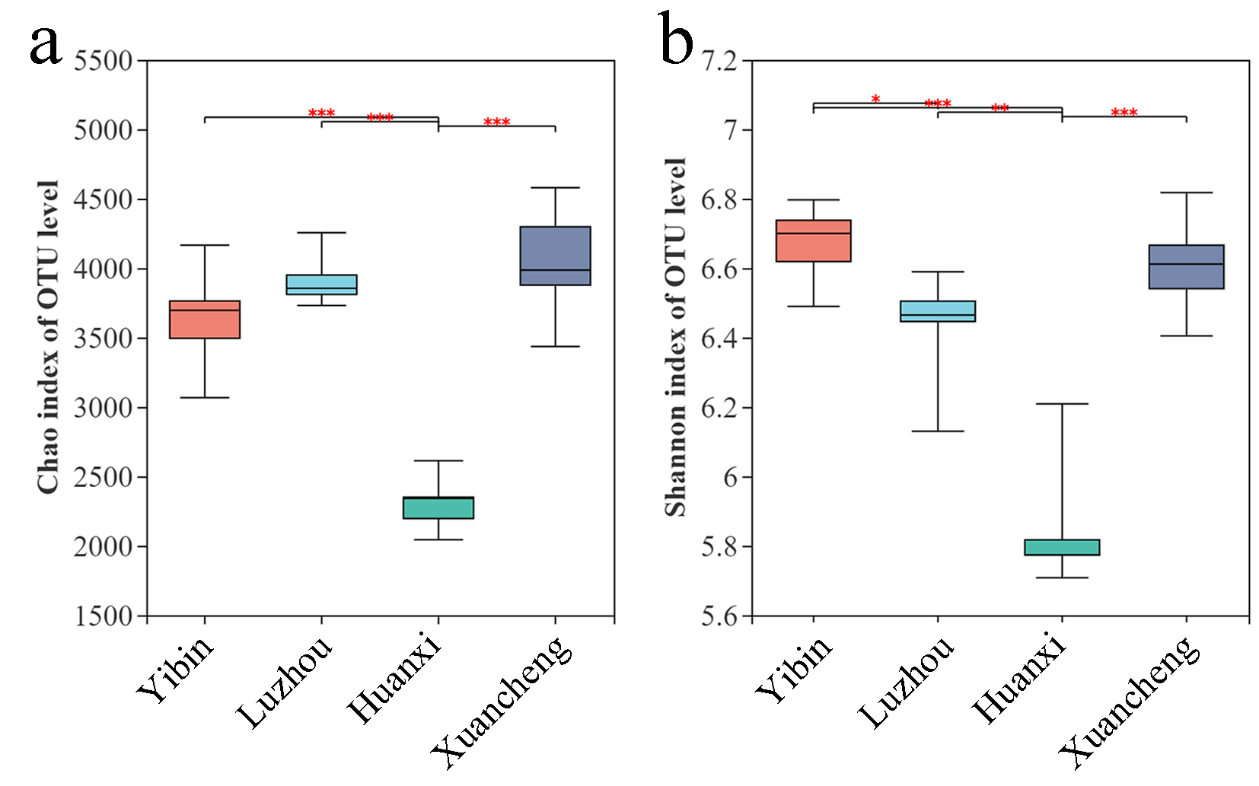


Figure S5. Comparison of Chao1 and Shannon of bacterial communities of BS between Yibin, Luzhou, Huanxi and Xuancheng. a. Comparison of Chao1 of bacterial communities of BS between Yibin, Luzhou, Huanxi and Xuancheng. b. Comparison of Shannon of bacterial communities of BS between Yibin, Luzhou, Huanxi and Shannon. Data were analyzed by student’ t-test (^*^: 0.01< *p* ≤ 0.05, ^**^: 0.001 < *p* ≤ 0.01, ^***^: *p* ≤ 0.001).
