# Supplementary material for: Analysis of rhizosphere bacterial communities of tobacco resistant and non-resistant to bacterial wilt in different regions
Source: Sci Rep. 2022 Oct 31;12:18309. doi: 10.1038/s41598-022-20293-6 (PMC9622857; doi:10.1038/s41598-022-20293-6)
Supplement: Supplementary file 7 — Supplementary Table S2. [file 41598_2022_20293_MOESM7_ESM.docx]

Table S2. Number of positive and negative correlation networks for KS, GS and BS for Xuancheng, Luzhou, Huanxi and Yibin in the top 50 species.

| **Region** | **KS** | **GS** | **BS** |
| --- | --- | --- | --- |
| Xuancheng | 224 | 201 | 408 |
| Luzhou | 500 | 228 | 294 |
| Huanxi | 452 | 288 | 264 |
| Yibin | 286 | 314 | 248 |
