# Supplementary material for: Analysis of rhizosphere bacterial communities of tobacco resistant and non-resistant to bacterial wilt in different regions
Source: Sci Rep. 2022 Oct 31;12:18309. doi: 10.1038/s41598-022-20293-6 (PMC9622857; doi:10.1038/s41598-022-20293-6)
Supplement: Supplementary file 8 — Supplementary Table S3. [file 41598_2022_20293_MOESM8_ESM.docx]

Table S2. Interaction counts of the top 50 bacterial communities in terms of KS and GS abundance in Yibin, Luzhou, Huanxi and Xuancheng.

| **OTU** | **Genus** | **Yibin** | | **Luzhou** | | **Huanxi** | | **Xuancheng** | |
| --- | --- | --- | --- | --- | --- | --- | --- | --- | --- |
|  |  | **KS** | **GS** | **KS** | **GS** | **KS** | **GS** | **KS** | **GS** |
| OTU11002 | Micromonospora | 11 | 2 |  |  |  |  |  |  |
| OTU878 | Sphingomonas | 12 | 6 |  |  | 17 | 9 |  |  |
| OTU9742 | Sphingomonas |  |  | 18 | 1 |  |  |  |  |
| OTU9531 | Streptomyces |  |  | 11 | 3 |  |  |  |  |
| OTU878 | Sphingomonas |  |  | 17 | 5 |  |  |  |  |
| OTU6148 | Rhodanobacter |  |  | 11 | 3 |  |  |  |  |
